# Class switched bovine ultralong CDR H3 amplicons versus canonical in immune and non-immune tissues

**DOI:** 10.1007/s00251-026-01395-1

**Published:** 2026-02-23

**Authors:** Karina L. Hissen, Morgan Sheppard, Jeannine A. Ott, Kerstin K. Landrock, Casey Whitley, Nicholas Ebert, J. M. Cody Horton, Nicole A. Glenn, Yoonsung Jung, Vaughn V. Smider, Michael F. Criscitiello

**Affiliations:** 1 Comparative Immunogenetics Lab, Department of Veterinary Pathobiology, College of Veterinary Medicine and Biomedical Sciences, Texas A&M University, College Station, TX 77843 USA; 2 Statistical Consulting Center, Department of Statistics, Texas A&M University, College Station, TX 77843 USA; 3 Department of Molecular Medicine, The Scripps Research Institute, La Jolla, CA 92037 USA; 4 Applied Biomedical Science Institute, San Diego, CA 92127 USA; 5 Department of Microbial Pathogenesis and Immunology, College of Medicine, Texas A&M Health Science Center, Texas A&M University, Bryan, TX 77807 USA

**Keywords:** Cattle, Ultralong CDR H3, Isotype, B cells, Class switch

## Abstract

Within the bovine immune response, a distinct type of immunoglobulin (Ig), known as ultralong complementarity-determining region 3 (CDR3) antibodies, coexists alongside canonical Igs. These ultralong heavy chains (HC) feature a remarkable structure with a lengthened CDR3 of the heavy chain (CDR H3), allowing them to adopt either a smaller and more extended paratope than canonical antibodies. However, very little is known about the distribution of ultralong CDR H3s in cattle tissues and which isotypes they are ultimately associated with. This study analyzed and quantified the amplicons of the five immunoglobulin isotypes (IgM, IgD, IgG, IgA, and IgE) for both the canonical and ultralong CDR H3 throughout 24 *Bos taurus* tissues. IgH amplicons analysis identified an extensive repertoire of expressed canonical and ultralong CDR H3s of each isotype in most tissues. Ultralong cattle Igs were preferentially switched to the IgG isotype, especially in the medial retropharyngeal lymph node draining the immunization site. Additionally, B cells producing canonical Igs in bone marrow switched most to the IgG isotype. These data suggest that ultralong CDR H3 may be uniquely qualified to handle some antigens. A more comprehensive understanding of isotype and tissue-specific immune responses of these unusual antibodies can provide a deeper insight into their function within the overall cattle immune system, offering unique opportunities for innovations for in both bovine health and human immunotherapeutics.

**Supplementary Information:**

The online version contains supplementary material available at 10.1007/s00251-026-01395-1.

## Introduction

A crucial adaptation in the vertebrate immune system is the production of antibodies, also known as secreted immunoglobulins (Ig), to neutralize foreign bodies. B cells secrete Ig to respond to repeated antigenic exposure through neutralization, complement activation, opsonization and cellular cytotoxicity. Igs are composed of four linked peptide chains, two heavy chains (HC) and two light chains (LC), each containing constant and variable regions, forming two antigen binding sites. There are five Ig isotypes based on their HC class of constant domain gene segments used: µ, δ, γ, ε, and α encoding IgM, IgD, IgG, IgE, and IgA, respectively (Knight et al. 1988; Symons et al. 1989; Kacskovics and Butler 1996; Rabbani et al. 1997; Brown et al. 1997; Mousavi et al. 1997, 1998; Zhao et al. 2006). IgM is the first Ig expressed by a B cell, and mature naïve B cells can temporarily display both IgM and IgD receptors on their surface, as IgD is expressed without class switch recombination (Stavnezer 1996). IgM antibodies are predominantly found in the blood and, to a much lesser extent, in the lymph, while IgD is found on the membrane of B cells in extremely low concentrations. During an immune response, activated B cells undergo somatic hypermutation (SHM) to affinity mature their receptors for better antigen binding (Kocks and Rajewsky 1988; Di Noia and Neuberger 2007; Wang et al. 2013; Doria-Rose and Joyce 2015). In the same germinal center reaction, they undergo class switching to produce Igs of the distinct functional isotypes IgG, IgA, and IgE.

At each of an immunoglobulin’s two antigen binding sites there are six complementarity-determining regions (CDR), three from each LC and three from each HC. Each B cell forms a unique rearrangement of variable (V), diversity (D) and joining (J) gene segments to form these binding sites, creating a diverse repertoire of paratope structures that bind a vast array of epitopes (Li et al. 2004; Conticello et al. 2005). Within these CDRs, CDR3 of the HC (CDR H3) contains the greatest combinatorial and junctional diversity and is usually responsible for making the greatest contact with antigens. The exceptionally long CDR H3 of a subset of Ig used by *Bos* and *Bison* species have piqued our interest for further investigation (Ott et al. 2023).

This cattle Ig subset contains an ultralong (UL) CDR H3, that can reach 40–70 amino acids (aa) (Berens et al. 1997; Lopez et al. 1998; Saini et al. 1999; Walther et al. 2013). Comparatively, a human CDR H3 is typically 8–16 aa in length (Wang et al. 2013), while mouse, sheep, goat, camelids and pigs exhibited ranges of 5–26 aa (Zemlin et al. 2003; Wu et al. 2012; Shingai et al. 2024), 4–27 aa (Park et al. 2023; Wu et al. 2025), 9–23 aa (Du et al. 2018), 5–25 aa (Zhang et al. 2025), and 4–14 aa (Yamamoto et al. 2005), respectively. Interestingly, the bovine CDR H3 has a trimodal distribution where clusters are sometimes classified as short (≤ 10 aa), medium (11–39 aa), and ultralong CDR H3s (≥ 40 aa) (Walther et al. 2013, 2016; Oyola et al. 2021; Altvater-Hughes et al. 2024, 2025). These ultralong HCs can bind antigen epitopes with the single CDR H3 that often are inaccessible to more planar, six-CDR paratopes of canonical antibodies (Sok et al. 2017; Stanfield et al. 2016; Criscitiello 2021; Huang et al. 2023).

The ultralong HC CDR3 consists of a β-ribbon “stalk” and disulfide-linked “knob” (Wang et al. 2013). These stalk and knob domains form by recombining a specific germline V, one particular D, and one of two J gene segments into an elongated CDR H3. The β-ribbon ascending strand of the stalk is formed by an 8-bp duplication at the 3’ end of the IGHV1-7 segment, which extends the nucleotide sequence and encodes a TTVHQ (threonine-threonine-valine-histidine-glutamine) motif amino-terminal to the YYC (tyrosine-tyrosine-cysteine) typically found at the end of a canonical HC V region (Wang et al. 2013; Ott et al. 2023). This motif forms a critical β-ribbon ascending strand of the stalk domain. To form the β-turn at the base of the knob of a conserved CPDG (cysteine-proline-aspartic acid-glycine) motif found at the beginning of the IGHD8-2 (Wang et al. 2013). The IGHD segment encodes the entirety of the knob and the beginning of the descending β-ribbon strand of the stalk domain. Within the knob domain, repetitive codons for glycine (GGT), tyrosine (TAT), and serine (AGT) residues can be somatically mutated to cysteine (TGT) with a single base change, and rearranged loci undergo extensive SHM and even truncations catalyzed by activation-induced cytidine deaminase (AID) within the knob sequences, thereby altering the number of cysteine residues within the knob (Wang et al. 2013). These added and altered cysteines modify the disulfide patterns in the knob expanding the repertoire of this “picobody” (Wang et al. 2013; Deiss et al. 2019) and adding remarkable stability to the domain (Svilenov et al. 2021; Passon et al. 2025).

Much is known about the structure of ultralong “cattlebodies”, with studies revealing the likely evolutionary factors that underlie our current understanding of the mechanisms for generating diversity and functionality. The ultralong CDR H3 Ig do class switch (Walther et al. 2013); however, we do not fully understand the distribution of ultralong CDR H3 across cattle tissues post SHM, which could help to elucidate their overall function in the cattle immune system. To our knowledge, this is the first study to investigate the differential expression of ultralong and canonical HC amplicons in a diverse set of cattle tissues, specifically the relative amounts of ultralong Ig in pre-switch isotypes versus post-switch isotypes. We assessed rearranged Ig HC transcripts from various tissues of two hyperimmunized two-year-old steers for CDR3 and isotype used in ultralong and canonical rearrangements. With a more complete understanding of tissue-specific expression patterns and isotype-specific immune responses using these exceptional paratopes, we’ll be better poised to exploit them for improved bovine and human health.

## Methods

### Collection of tissue samples, isolation of RNA, and synthesis of cDNA

Tissues analyzed in this study were harvested from Brangus (Angus and Brahman cross) steers (*Bos taurus)* 1817 and 1851, housed and immunized at Texas A&M University Veterinary Medical Park under TAMU IACUC 2022−0059. The animals were immunized with two TIM-3 (T cell immunoglobulin and mucin domain 3) were given intradermally to prime and boost an immune response in steer 1817, and against PD-1 (programmed cell death 1 antigen) in steer 1851. Both immunogens were recombinantly produced extracellular domains of the human protein. While the two immunization strategies suggest different experiments, the data were drawn with distinct metadata contexts, allowing the evaluation of biological patterns that represent the natural range of responsiveness within this sample set. See Supplemental Fig. 1 for timeline, components of injections, and antibody titers from isolated peripheral blood confirming immunization through antigen-specific IgG enzyme-linked immunosorbent assays (ELISA). Briefly, 96-well plates (BD Biosciences, San Jose, CA) were coated with 100 µL of 1 ng/µL antigen in filtered, autoclaved coating buffer at pH 9.6, containing 2.93 g NaHCO3 and 1.5 g Na2CO3 in 1000mL dH2O. The plates were then sealed and incubated overnight at 4℃. The next day, the plates were decanted and blotted on a paper towel three times before washing the wells five times with 200 µL of room-temperature 1X Tris-Buffered Saline (TBS) containing 0.1% Tween 20 (TBST). Next, 200 µL of blocking buffer consisting of 10% filtered equine whole sera in 1X TBST. The plates were then sealed and incubated at 37℃ for 60 min. Plates were washed five times as previously described before incubating each well with 150 µl of the respective diluted bovine serum or blocking buffer (background well). The plates were then sealed and incubated at 37℃ for 120 min. Thereafter, the wells were washed five times as described earlier before placing 150 µl of 1:4000 Peroxidase-conjugated AffiniPure goat anti-bovine IgG (H + L) (Jackson ImmunoResearch Laboratories, West Grove, PA, USA) or 150 µl of the blocking buffer. The plates were then sealed and incubated at 37℃ for 60 min. Afterwards, the wells were washed four times with 200µL of room temperature 1X TBST, then washed three times with 200µL of room temperature 1X TBS. Subsequently, 150µL of ready-to-use 3,3′,5,5′-Tetramethylbenzidine substrate was added to each well and incubated at room temperature for 10 min or until a blue color appears. If no color develops after 10 min, further incubation was performed at 37℃ and reassessed periodically. Once developed, the reaction was stopped with 150µL of 1 M H_2_SO_4_ and then read on a OD-450 plate reader.

**Fig. 1:**
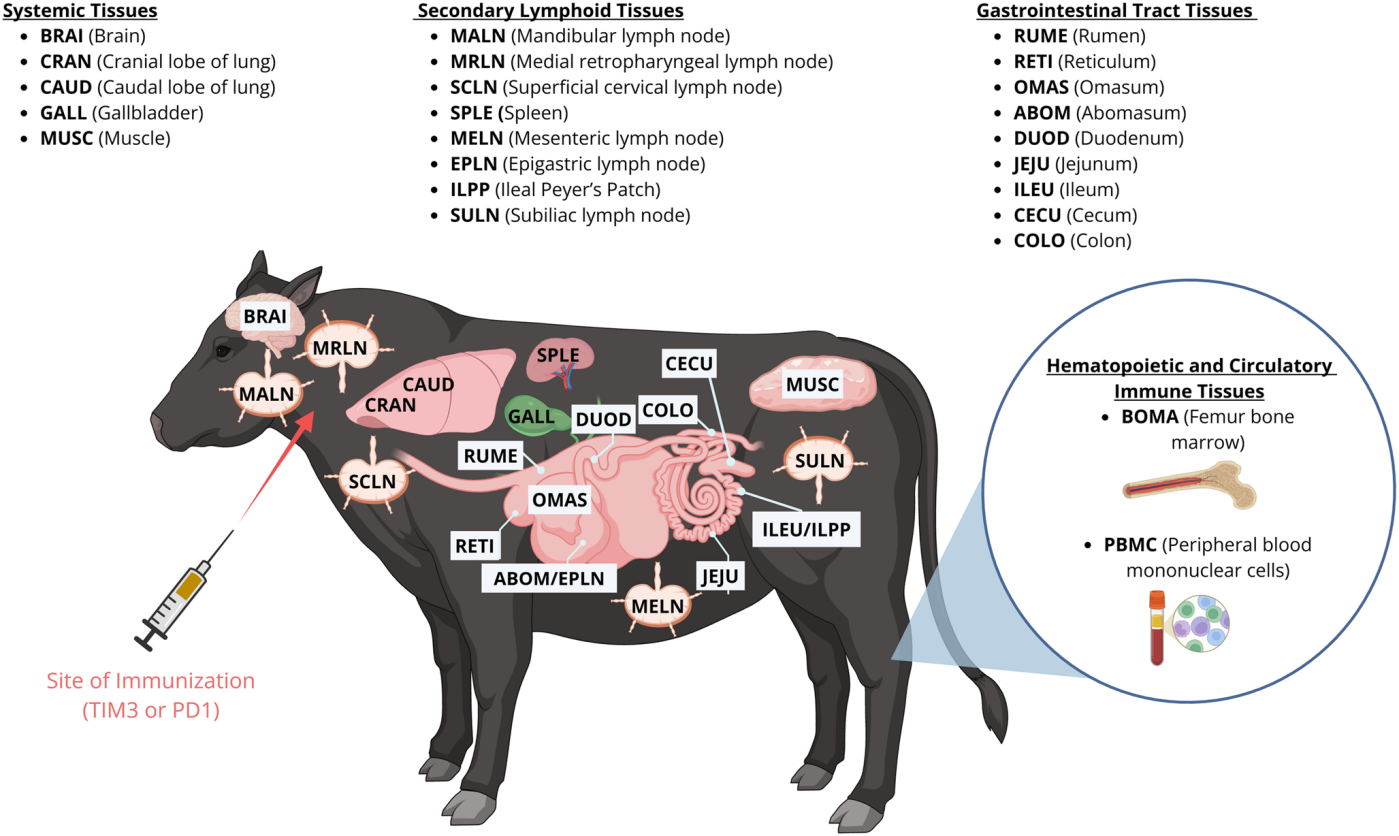
Tissues collected from steers. Tissues are in order from anterior to posterior based on four categories (systemic, secondary lymphoid tissues, gastrointestinal tract, and hematopoietic and circulatory immune tissues). Each tissue has been assigned a four-letter acronym. Created in BioRender. Hissen, K. (2026) https://BioRender.com/cn2orzb

Figure 1 depicts the 24 tissues collected from both steers in order from anterior to posterior based on four major tissue categories: systemic, secondary lymphoid, gastrointestinal tract, hematopoietic, and circulatory immune tissues. Tissues are listed as follows alphabetically with an unique acronym: abomasum (ABOM), bone marrow (BOMA), brain (BRAI), caudal lobe of the lung (CAUD), cecum (CECU), colon (COLO), cranial lobe of the lung (CRAN), duodenum (DUOD), epigastric lymph node (EPLN), gallbladder (GALL), ileum (ILEU), ileal Peyer’s patch (ILPP), jejunum (JEJU), mandibular lymph node (MALN), mesenteric lymph node (MELN), medial retropharyngeal lymph node (MRLN), muscle (MUSC), omasum (OMAS), peripheral blood mononuclear cells (PBMC), reticulum (RETI), rumen (RUME), superficial cervical lymph node (SCLN), spleen (SPLE), and subiliac lymph node (SULN). Once collected, all tissues were preserved for 24 h in RNAlater (ThermoFisher Scientific, Waltham, MA, USA) at 4 °C and then stored at −20 °C until processing.

Total RNA was purified from each tissue using Ambion TRIzol reagent (ThermoFisher Scientific) following the manufacturer’s instructions. Briefly, about 100 mg of tissue was homogenized in TRIzol reagent using a Tissuelyser II (Qiagen, Hilden, Germany). Additionally, 9.0 × 10^6^ (steer 1817) and 3.3 × 10^6^ (steer 1851) PBMCs were suspended in TRIzol and homogenized by passing the suspension through a 20-gauge 1.5” needle attached to a 1 mL syringe (BD, Franklin Lakes, NJ, USA). RNA quantity (ng/µl) and quality (260/280) were measured using a Nanodrop^®^ ND-1000 spectrophotometer (ThermoFisher Scientific).

Using the mRNA, cDNA was synthesized using SuperScript III First-Stand System (Invitrogen, Waltham, MA, USA) reverse transcriptase following the manufacturer’s instructions with 5 µg of RNA. Both quantity and quality of the synthesized cDNA were measured using a Nanodrop^®^. The cDNA concentrations were standardized from all tissues to 400 ng/µl for PCR reactions.

### Amplification of canonical and ultralong CDR H3 sequences

Using Geneious Prime 2022.0.1 (https://www.geneious.com), a single forward primer was designed to a conserved region of both IGHV1-7 (ultralong HC V) and IGHV1-10 (canonical HC V), for ultralong CDR H3 arises from multiple VH families. Reverse primers were designed to the CH1 domain exon of IgM, IgD, IgG, IgE, and IgA constant regions (Walther et al. 2013. Deiss et al. 2019). Primers were tested both individually and as a multiplex of all five reverse primers using pooled calf cDNA (as above), choosing to use a multiplex of the reverse primers in subsequent reactions. DreamTaq PCR master mix (Invitrogen) was used to amplify target sequences and PCR products were visualized using a 1.5% agarose gel to confirm band sizes (Supplemental Fig. 2). Each primer was then appended with partial Illumina adaptors for sequencing. Unique barcodes of eight base pair (bp) in length were created and inserted between the P5 Illumina adaptor and the forward primer sequence (P5 – barcode – primer) to create primers designed to distinguish sequenced amplicons by tissue (Supplemental Table 1). This second set of primers was retested using the same process as above, in addition to Phusion High-Fidelity PCR Master Mix polymerase (ThermoFisher Scientific).

To obtain IgH VH-CH1 amplicon sequences from each tissue, a final 50 µl PCR reaction was ran using 400 ng cDNA, primers (5 µM forward and a 5 µM reverse primer mix) containing adaptors and barcodes, and Phusion High-Fidelity PCR Master Mix with the following cycling parameters: 30 s at 98 °C (initial denaturation), 30 cycles of 30 s at 98 °C (denaturation), 30 s at 58 °C (annealing), and 60 s at 72 °C (extension), with a final extension of 10 min at 72 °C. After PCR amplification, the presence of resulting bands was confirmed and amplicon lengths were validated using a 1% agarose gel (run for 75 min at 83 V using 5 µl GelGreen, 1 µl GeneRuler 1 kb Plus DNA Ladder (ThermoFisher Scientific), and 5 µl of the sample mixed with 1 µl of 6x loading buffer) as shown in Supplemental Figs. 3 and 4. ExoSAP-IT Express reagent (ThermoFisher Scientific) was used to purify the remaining PCR product (45 µl) enzymatically, then the amplicon was measured for DNA concentration using a Qubit 4 fluorometer (Invitrogen). These targets were confirmed through sequencing.

### Amplicon sequencing and bioinformatic analysis

We submitted a pooled sample containing 2 µl of each tissue PCR product for amplicon sequencing (Amplicon-EZ Illumina-based sequencing services; Azenta Life Sciences, South Plainfield, NJ, USA). A total of 566,628 paired reads were obtained via Amplicon-EZ sequencing. Using default options of the BBMerge paired read merger in Geneious Prime (Biomatters, Ltd, Auckland, New Zealand, v2023.1.1), the reads were merged into 180,787 sequences and sorted by barcode, allowing a single mismatch in the barcode sequence. The tissue indicated by the barcode was appended to sequence names. VDJ-C germline reference sequences were created for each isotype by concatenating germline V (canonical IGHV1-10; ultralong IGHV1-7), D (canonical IGHD6-2*02; ultralong IGHD8-2*02), and J (IGHJ2-04) germline gene segments with constant regions for IgM, IgD, IgG, IgE, and IgA. Simultaneously, the 180,787 sequences were mapped to Ig reference sequences for both the canonical HC and ultralong HC using default mapper settings in Geneious Prime. Geneious mapper assembled 165,374 reads to two reference sequences to create two contigs.

**Fig. 2:**
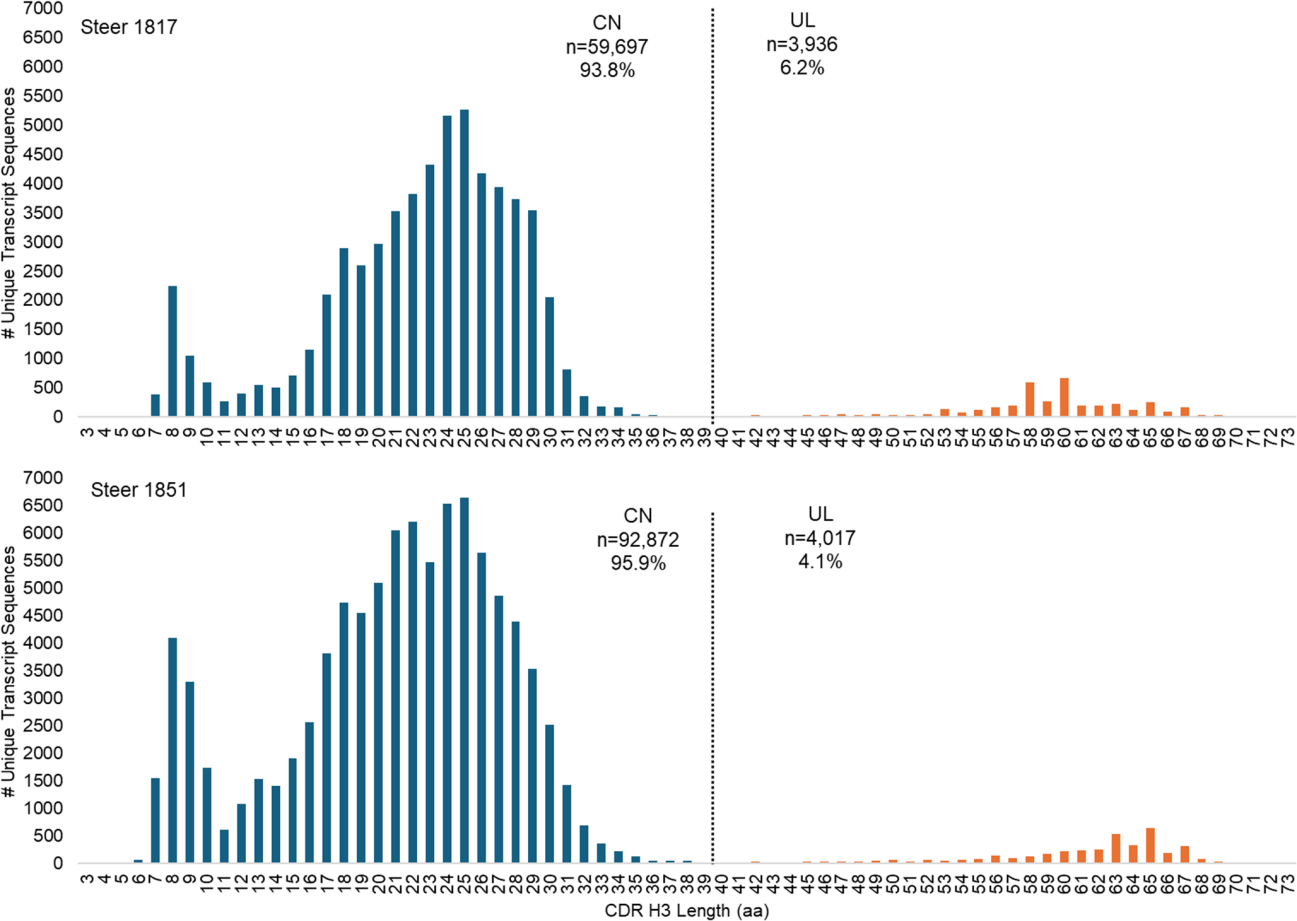
Steers share similar patterns of CDR H3 amino acid (aa) length. Within the canonical (CN, blue) CDR H3, there was a shoulder peak consisting of ≤ 11 aa, in addition to the main peak 12–39 aa in length. Ultralong (UL, orange) CDR H3s were defined as anything ≥ 40 aa in length

We confirmed that each contig contained either the canonical HC or ultralong HC sequences using NCBI BLASTn (Zhang et al. 2000) and validated all amplicons within a contig to ensure they contained either the canonical HC or ultralong HC sequences by searching for the encoded YYC motif typical of canonical (IGHV1-10) or the 8-bp duplication encoding the TTVHQ motif found in ultralong CDR H3 sequences (IGHV1-7). For this study both the very short CDR H3s peaking at 8 aa and the dominant population peaking at 25 aa up until 39 aa are all considered canonical (Fig. 2). We extracted the nucleotide sequence from each contig between the encoded aspartic acid residue (D) at position 98 (DXATYYC) of the V segment and residue 24 of the C region. We then reassembled sequences to specific germline reference sequences for all five isotypes, creating five contigs each for canonical HC and ultralong HC amplicons. We extracted all sequences greater than 150 bp in length and validated isotypes within each contig using the constant region sequences, moving sequences to appropriate isotype files as necessary. Finally, we aligned canonical HC or ultralong HC sequences by isotype, and appended to the sequence name for analysis. We have provided our sequences in Excel files for each steer within the supplementary data. Each file contains two tabs of the raw, unfiltered output that was exported. The functional tab includes sequences that have not been aligned to the canonical HC or ultralong HC but meet the criteria indicated above. The unique tab contains the sequences that have been aligned to canonical HC or ultralong HC and was used for data analysis.

### Statistical analysis

All statistical analyses were performed using SAS Analytics software (version 9.4) and JMP Pro 17 software (SAS Institute Cary, NC). A paired t-test was used to compare the proportions of unswitched and switched isotypes in each tissue across different CDR H3 lengths. Factorial analyses of variance (ANOVA) were used to evaluate the effects of CDR H3 length, immunoglobulin isotype, and tissue on response variables. Three-way, two-way, and one-way ANOVA models were fitted as appropriate to the experimental design with a significance of α = 0.05. When the overall ANOVA was significant (α = 0.05), pairwise comparisons among group means were performed using Tukey’s honestly significant difference (HSD) post hoc test.

## Results

### Length distribution of CDR H3

The distribution of canonical and ultralong CDR H3 aa lengths (denoted as CN and UL in figures, respectively) of unique transcript sequences was remarkably similar between both steers across all isotypes and tissues examined. CDR H3 lengths showed a trimodal distribution, with a shoulder peak consisting of CDR H3 ≤ 11 aa a main peak of 12–39 aa, and an ultralong peak with CDR H3 length ≥ 40 aa (Fig. 2). The average length of all CDR H3 was 24.6 aa (steer 1817; *n* = 63,633 transcripts) and 22.6 (steer 1851; *n* = 96,889 transcripts). The average length of canonical CDR H3 was 22.4 aa (steer 1817) and 20.9 aa (steer 1851) and of ultralong CDR H3 was 58.9 aa (steer 1817) and 60.8 aa (steer 1851). Both steers shared similar patterns of VH, DH, and JH segment usage (Supplemental Fig. 5), with majority ultralong CDR H3 amplicons utilizing VH1-7, the gene segment containing the 8-base pair duplication that encodes the TTVHQ motif (1817: 95.8%; 1851: 94.0%). Additionally, ultralong CDR H3 utilized DH8-2 (1817: 37.8%; 1851: 34.1%), which contains a high density of germline encoded cysteines that contribute to the stalk and knob, and nearly all used JH2-4 (1817: 99.9%; 1851: 100%). The JH2-4 was also predominantly expressed in canonical CDR H3s. Our analysis likely discounts the use of DH8-2 in the ultralong CDR H3 as the extensive SHM and truncations thwart alignment with the germline D segment and afford matches to other shorter ones.

## Distribution of isotype

**Fig. 3:**
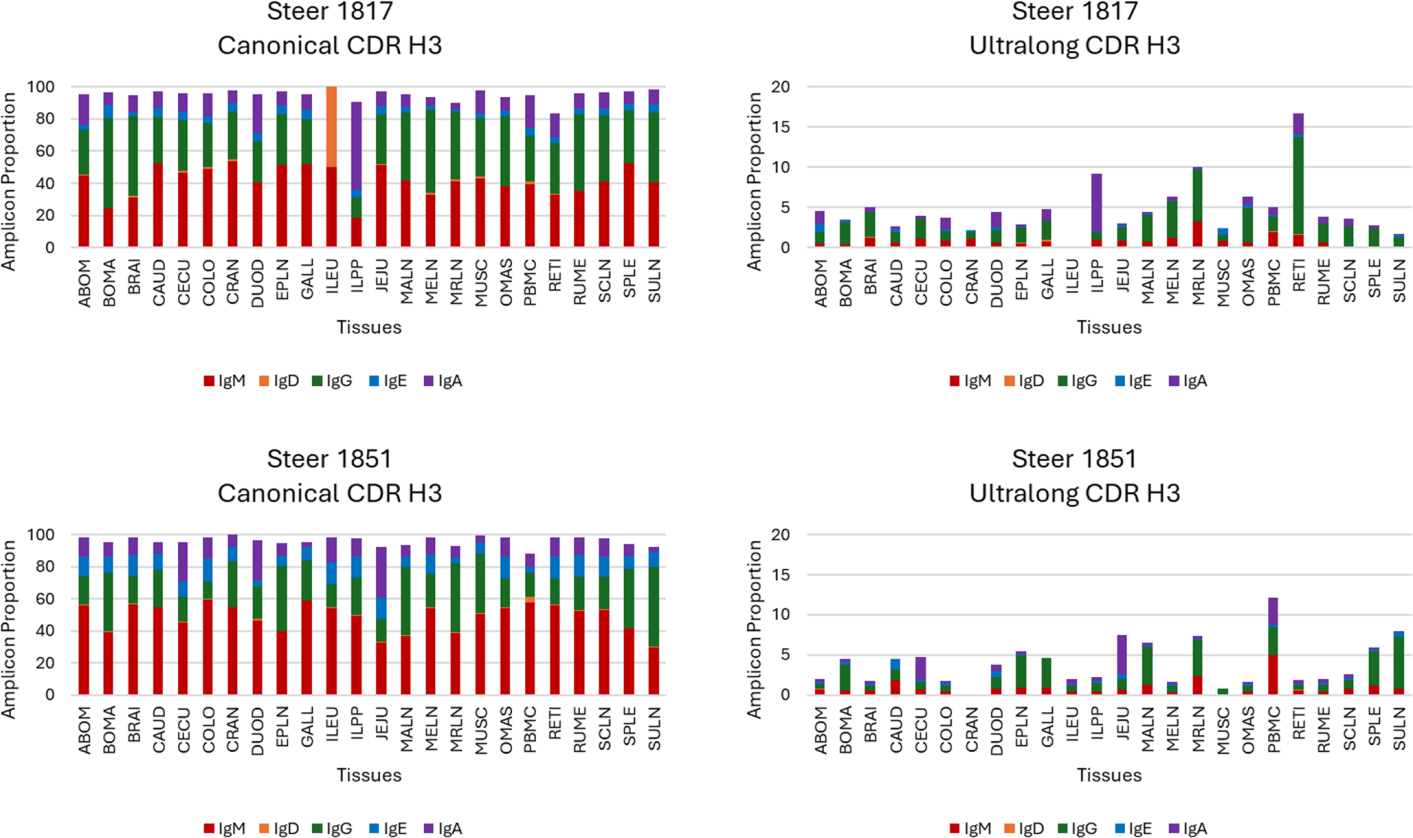
Steers show similar patterns of amplicon proportions across tissues within each CDR H3 length. Isotypes include IgM (red), IgD (orange), IgG (green), IgE (blue), and IgA (purple). Proportions were calculated for each tissue by taking amplicon counts for each isotype within CDR H3 length, then dividing by the total amplicon counts for in that tissue. Overall, canonical CDR H3 had a much higher proportion than ultralongs, particularly in the IgM and IgG isotypes

The distribution of the five isotypes were similar between the two steers across all tissues within both canonical and ultralong CDR H3 lengths (Supplemental Fig. 6). Additionally, steers show similar patterns of amplicon proportions across tissues within each CDR H3 length. Overall, canonical CDR H3 had a much higher proportion than ultralongs, particularly in the IgM and IgG isotypes (Fig. 3; Table 1). Few tissues exhibited differences in proportions. Steer 1817 had only 2 amplicon counts in the ileum (ILEU) within the canonical CDR H3, one for IgM and the other for IgD, indicating a higher proportion of IgD (Supplemental Table 2). Among the ultralong CDR H3s, steer 1817 exhibited higher proportions of IgG in the omasum (OMAS), reticulum (RETI) and IgA in the ileum Peyer’s path (ILPP) while steer 1851 had higher proportions of IgA in the jejunum (JEJU) and PMBCs, in addition to higher proportions of IgG in PMBCs, spleen (SPLE), and subiliac lymph node (SULN), as shown in Fig. 3; Table 1. This is due to differences in the total amplicon counts in these tissues between the two steers (Supplemental Table 2). Most importantly, these are proportions and asides counts for the ileum of steer 1817 were 2, and the cranial lobe of the lung (CRAN) of steer 1851 were 79; the rest were > 100, as shown in Supplemental Table 2.

**Table 1:**
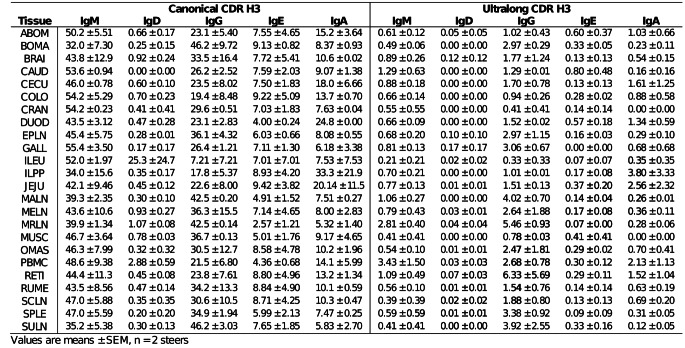
Steers amplicon proportion in each tissue within each CDR H3 length. Proportions were calculated for each tissue by taking amplicon counts for each isotype within CDR H3 length, then dividing by the total amplicon counts for in that tissue

**Fig. 4:**
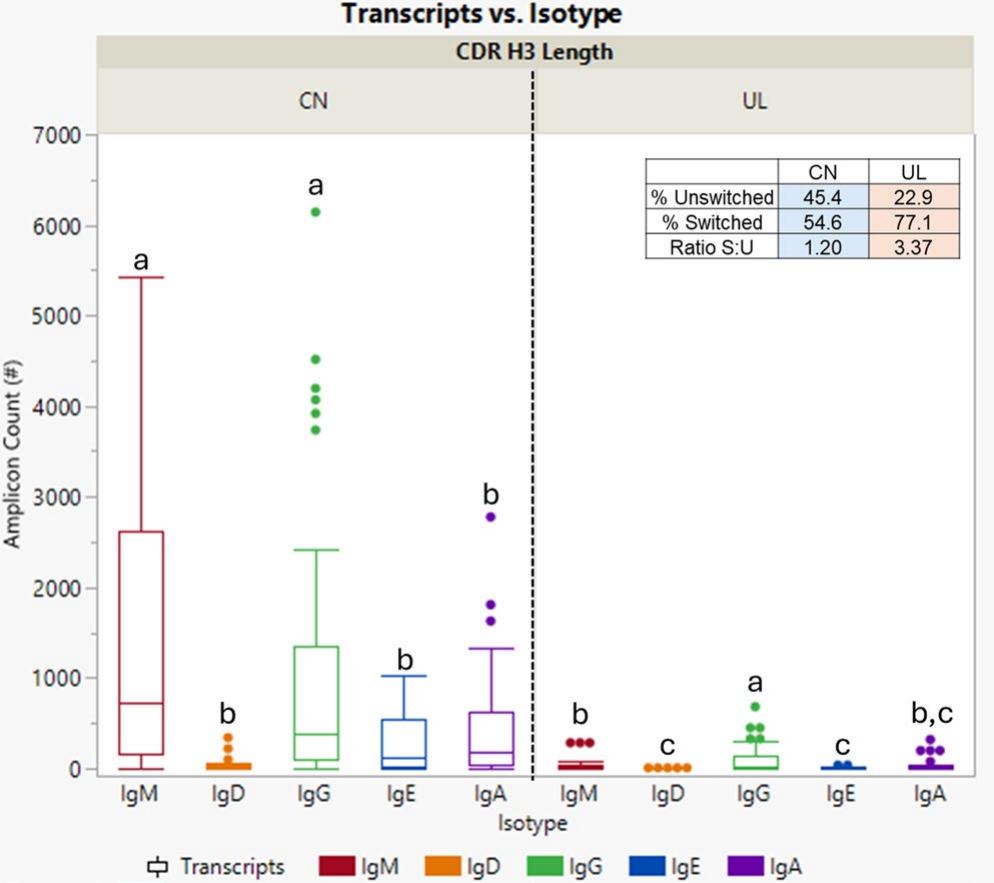
Ultralong Igs are preferentially switched to the IgG isotype. Unswitched isotypes include IgM (red) and IgD (orange), while switched isotypes include IgG (green), IgE (blue), and IgA (purple). Between steers, the IgM and IgG isotypes were the dominant Igs found within the canonical CDR H3 length (*P* < 0.0001). In contrast, most Igs within the ultralong CDR H3 lengths were preferentially switched to the IgG isotype (*P* < 0.0001)

Among the unswitched isotypes, IgM was more abundant than IgD. In switched isotypes, IgG use was greater than either IgE or IgA. For both steers, IgM and IgG isotypes comprised the majority of amplicons within the canonical CDR H3 (*P* < 0.0001), while most ultralong CDR H3 amplicons were preferentially switched to IgG (*P* < 0.0001; Fig. 4). There was a significant interaction of CDR H3 length and isotype on amplicon counts (*P* < 0.0001), as well as independently (*P* < 0.0001). IgM and IgG isotypes were the most abundant canonical amplicons compared to all ultralong isotypes (multiple comparison data not shown). The table within Fig. 4 demonstrates the overall switched-to-unswitched ratio. The two steers’ canonical Igs class-switched isotypes averaged 54.6%, while the ultralong Igs were 77.1% switched. The ratio and percentage were calculated based on the understanding that unswitched isotypes include IgM and IgD, while switched isotypes include IgG, IgE, and IgA. Amplicon counts were significantly higher in canonical Igs than in ultralong Igs (*P* < 0.0001).

### Percentage of cysteines within sequences was generally higher in ultralong CDR H3

Overall, ultralong CDR H3 had a higher cysteine percentage than the canonical CDR H3 at 1.3%, as shown in Table 2. Additionally, ultralong CDR H3 had a higher percentage of cysteine than the canonical CDR H3 across tissues, except in the cranial lobe of the lung (CRAN), where canonical CDR H3 had 0.6% more cysteines. The largest difference in cysteine percentage was observed in the caudal lobe of the lung (CAUD), followed by PMBCs, with 2.0% and 1.7% more in ultralong CDR H3, respectively. Importantly, both datasets are inflated by the intradomain disulfide forming cysteine at the carboxy end of the variable, which by convention is counted in each dataset.

**Table 2:**
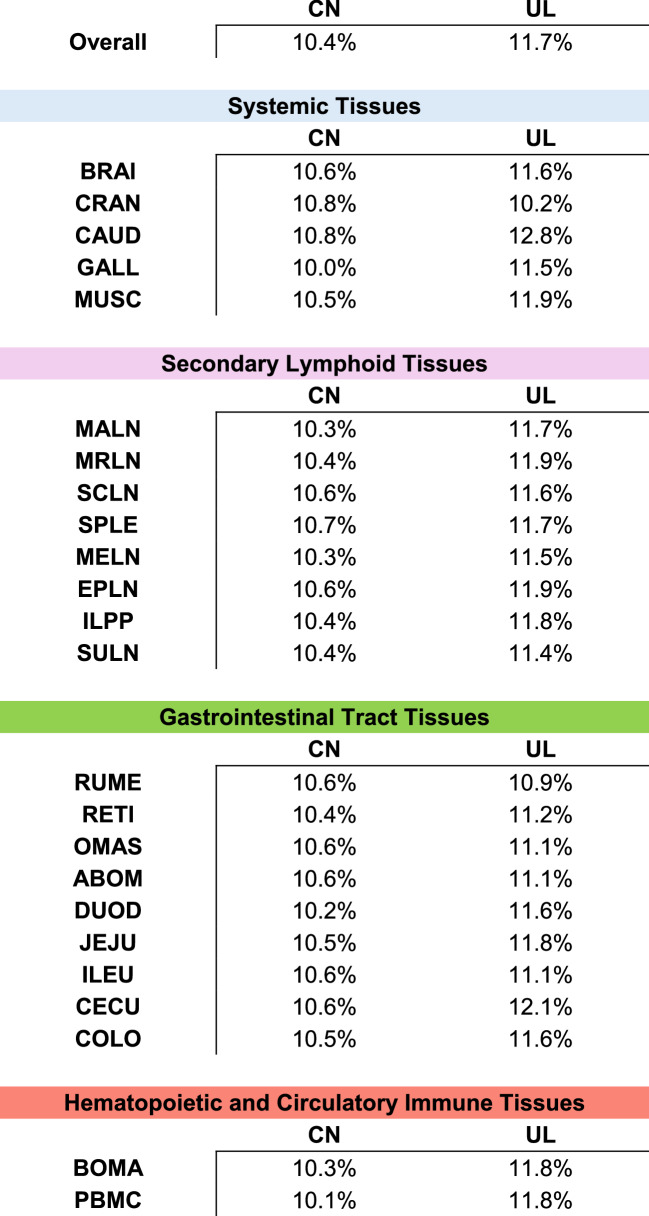
The percentage of cysteines within sequences were generally higher in ultralong (UL) CDR H3. Overall, UL CDR H3 had a higher cysteine percentage than the canonical (CN) CDR H3 at 1.3%. Additionally, UL CDR H3 had a higher cysteine percentage than the canonical CDR H3 across tissues, except in the cranial lobe of the lung (CRAN) where CN CDR H3 had 0.6% more cysteines. The largest difference in cysteine percentage was observed in the caudal lobe of the lung (CAUD), followed by PMBCs, with 2.0% and 1.7% more in UL CDR H3, respectively

### Class switching across tissues

**Fig. 5:**
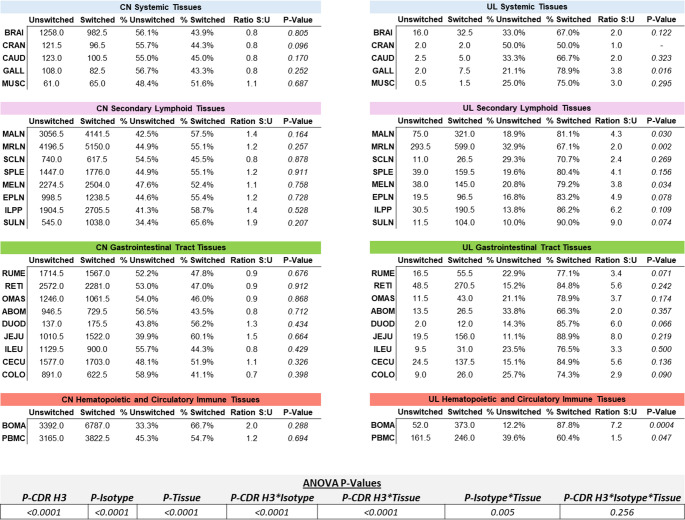
Ultralong Igs had a proportionally higher number of switched isotypes across all tissues. Tissues are categorized into four main groups, ordered from anterior to posterior. P-values for each tissue within each CDR H3 length are provided based on a paired t-test to compare proportions of unswitched and switched isotypes. Ratios were calculated based on proportions of switched to unswitched antibodies

Ultralong Igs had a relatively higher number of class switched transcripts across all tissues compared to that of the canonical CDR H3 length (Fig. 5). Ratios were calculated based on proportions of switched to unswitched Ig. The bone marrow (BOMA) had a significantly higher ratio of switched to unswitched Ig at 7.2 (*P* = 0.0004). Other noteworthy tissues include the gallbladder at 3.8 (GALL; *P* = 0.016), mandibular lymph node at 4.3 (MALN; *P* = 0.030), medial retropharyngeal lymph node at 2.0 (MRLN; *P* = 0.002), mesenteric lymph node at 3.8 (MELN; *P* = 0.034), and peripheral blood mononuclear cells at 1.5 (PBMC; *P* = 0.047). In the cranial lobe of the lung (CRAN), the proportion of switched and unswitched isotypes was identical; thus, no test statistic could be computed, indicating no difference between means. ANOVA analysis revealed that while the full factorial effects of CDR H3 length, isotype, and tissue did not show significance (*P* = 0.256), there were interaction effects between isotype and tissues (*P* < 0.005). Additionally, there was statistical evidence of interaction effects with CDR H3 length and isotype (*P* < 0.0001) as well as CDR H3 length and tissue (*P* < 0.0001).

### Interaction effects of CDR H3 in tissues

**Fig. 6:**
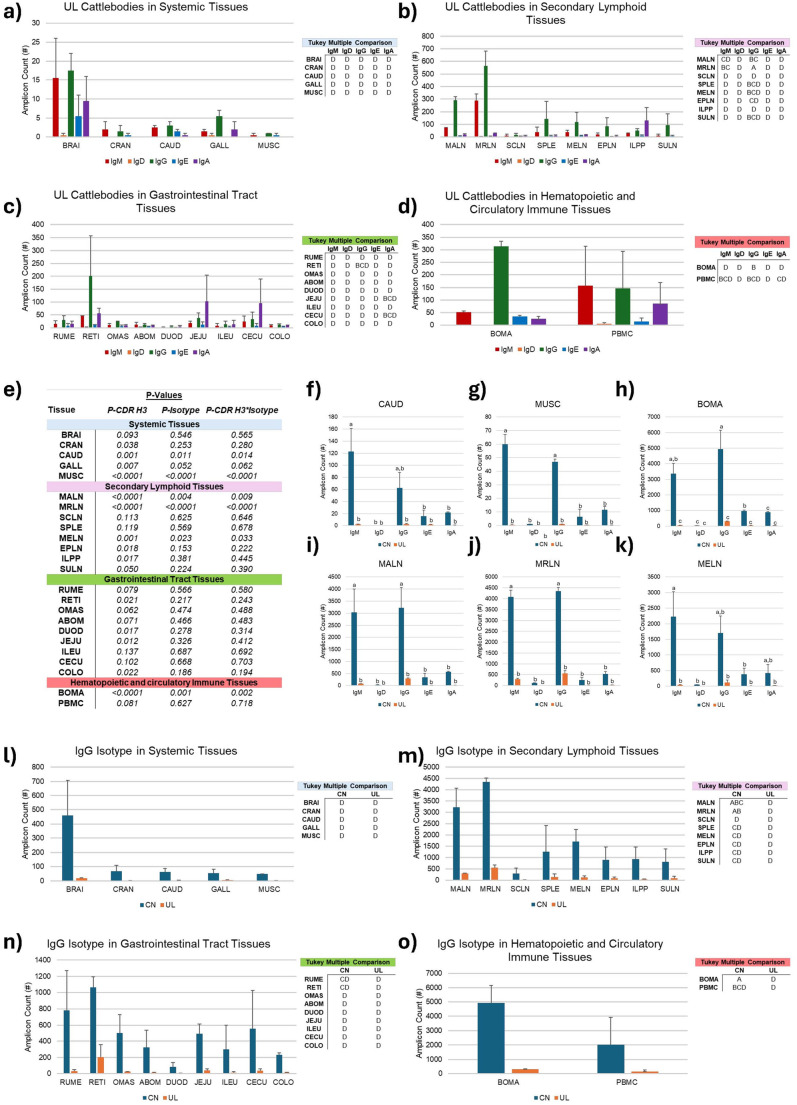
Interaction effects of CDR H3 in tissues among both steers. [a-d] The first analysis examined isotype and tissue effects by CDR H3 length, with notable effects observed within the ultralong CDR H3 length (P < 0.0001). Tissues are grouped into four main categories, showing their amplicon counts in unswitched isotypes, IgM (red) and IgD (orange), and switched isotypes, IgG (green), IgE (blue), and IgA (purple). Tables display letters a-d, where means not sharing a letter differ (P < 0.05) as determined by two-way ANOVA and the Tukey multiple comparison test. [e-k] The second analysis inspected CDR H3 length and isotype across tissues, revealing strong effects observed in six tissues (P < 0.0001), while the remaining 18 tissues did not show significant interaction effects (Fig. 5e). In the figures, amplicon counts are shown for each CDR H3 length, categorized as canonical (CN; blue) and ultralong (UL; orange). Within each tissue, each bar is labeled with a letter, a-d, where a-d means that the bars without a common letter differ (P < 0.05), as determined by two-way ANOVA and the Tukey multiple comparison test. [l-o] The final analysis surveyed CDR H3 length and tissue effects by isotype, with significant interaction observed only within the IgG isotype (P < 0.0001). Tissues are grouped into four main categories, where amplicon counts are shown for each CDR H3 length, categorized as canonical (CN; blue) and ultralong (UL; orange). Tables display letters a-d, indicating that means without a common letter significantly differ (P < 0.05) based on two-way ANOVA and Tukey’s multiple comparison test

Interaction effects of CDR H3 length, isotype and tissue were analyzed for these Ig amplicons (Fig. 6). The first analysis investigated isotype and tissue effects by CDR H3 length, where notable effects were seen within the ultralong CDR H3 length (*P* < 0.0001). Shown in Fig. 5b, the medial retropharyngeal lymph node has the most total amplicons for all isotypes, where the IgG isotype had the most amplicon counts compared to any other isotype and tissue combination (563 ± 118). There were no interaction effects seen within the canonical CDR H3 length (*P* = 0.079). The bone marrow had the next highest counts (314 ± 20), but it did not statistically differ from the IgA isotype within the cecum (CECU; 95 ± 94), IgA in the ileal Peyer’s patch (ILPP; 132 ± 102), IgA in the jejunum (JEJU; 104 ± 101), IgG in the reticulum (RETI; 202 ± 155), IgG in the spleen (SPLE; 144.5 ± 137.5), IgG in the subiliac lymph node (SULN; 95 ± 87), IgG in the mandibular lymph node (291 ± 28), IgG in the mesenteric lymph node (MELN; 119 ± 77), IgM in the medial retropharyngeal lymph node (289.5 ± 52.5), IgG and IgM in peripheral blood mononuclear cells (147 ± 114; 157 ± 110) as shown Fig. 6b-d.

The second analysis tested CDR H3 length and isotype by tissue, where robust effects were seen in six tissues (*P* < 0.0001), while the remaining 18 tissues did not display significant interaction effects (Fig. 5e). Regardless of tissue, canonical Igs had higher counts compared to that of ultralong Igs. Among the systemic tissues, the caudal lobe of the lung (CAUD; Fig. 6f) had more counts of the IgM isotype (123.0 ± 38.0) compared to all ultralong CDR H3 isotype combinations (*P* = 0.014). However, it was statistically similar to the IgG isotype (62.5 ± 25.5). Additionally, the muscle (MUSC; Fig. 5g) had substantially higher counts in both the IgM isotype (60.7 ± 7.0) and IgG isotype (47.0 ± 2.0) than all ultralong and other canonical isotype combinations (*P* < 0.0001). Across the immune tissues where B cells would be expected, the IgM and IgG amplicon counts were approximately 15–30 times higher compared to the caudal lobe of the lung and muscle. In the bone marrow (Fig. 5h), the IgM isotype had the most counts out of all CDR H3 isotype combinations (4937.5 ± 1204.5; *P* = 0.002) but did not statistically differ from the IgG isotype (3365.5 ± 653.5). Among the lymph nodes, the mandibular lymph node (Fig. 5i) had the highest expression in both the IgM (3032.0 ± 972.0) and IgG (3223.5 ± 843.5) isotypes than any other CDR H3 isotype combinations (*P* = 0.009). Similarly, the medial retropharyngeal lymph node (Fig. 5j) had significantly higher expression in both the IgM (4087.0 ± 301.0) and IgG (4353.5 ± 160.5) isotypes, which had considerably more counts than any other CDR H3 isotype combinations (*P* < 0.0001). Lastly, in the mesenteric lymph node (Fig. 5k), the IgM isotype had the most counts out of all CDR H3 isotype combinations (2230.0 ± 801.0; *P* = 0.033) but did not differ from the IgG isotype (1706.0 ± 541.0).

The last analysis examined CDR H3 length and tissue effects by isotype, where the significance of interaction was only seen within the IgG isotype (*P* < 0.0001). IgM, IgD, IgE, and IgA did not display statistically significant interaction effects (*P* = 0.294, 0.108, 0.663, and 0.673). Overall, the canonical CDR H3 length had more transcripts within the IgG isotype, with significantly higher counts in the bone marrow (4937.5 ± 1204.5; Fig. 5o). However, as shown in Fig. 5m, there was no statistical difference between counts in the bone marrow, mandibular lymph node (3223.5 ± 843.5), and medial retropharyngeal lymph node (4353.5 ± 160.5).

## Discussion

This deep tissue characterization of antibody expression in to hyperimmunized Brangus steers shows diverse isotype use throughout the sampled organs, ubiquitous ultralong CDR H3 Ig though at lower levels than canonical, and a predisposition for the ultralong CDR H3 Ig to use IgG, IgA and IgE suggestive of germinal center history.

A remarkable series of evolutionary innovations enable the immunogenetic diversification of vertebrate lymphocyte antigen receptors, including the use of AID-APOBEC family members for the earliest Variable Lymphocyte Receptor (VLR) diversification in agnathans (Rogozin et al. 2007) to the insertion of the transposon (Zhang et al. 2019) into *UrIg2* (Flajnik et al. 2025) for the birth of the RAG somatic recombination system. Much later, a fortuitous strand-slippage induced an eight-base pair duplication at the end of a variable segment in a Bovidae ancestor (Ott et al. 2023) and extension and AID recruitment of a diversity segment afforded the ultralong CDR3 (Deiss et al. 2019; Haakenson et al. 2019). Now attention is turned from the antibody structure and immunogenetics and into the animal and the potential physiology of the ultralong CDR H3 in bovine immunity.

### Length and VDJ usage

The CDR H3 length data presented here align with Altvater-Hughs et al., (2024) where a trimodal pattern was identified with canonical peaks focused at 8 and 25 aa in length, while ULs peaked at 60 and 65 aa in length for steer 1817 and 1851, respectively. Overall, there were a great many more canonical Ig amplicon counts, making it the bulk of the repertoire. This could be due to differential rearrangement and selection in the primary repertoire, or post-antigen repertoire skewing. The greater energy requirement for these longer structures suggests utility overcoming the cost. The majority of the ultralong Ig amplicons had gene segments utilizing VH1-7, DH8-2, and JH2-4 (Supplemental Fig. 5) as shown in Deiss et al. (2019) and Altvater-Hughs et al., (2024) (Deiss et al. 2019; Altvater-Hughes et al. 2024). The importance of this segment utilization in ultralong Igs was originally shown in a study on calves vaccinated against bovine respiratory disease (Wang et al. 2013). In addition to the segment utilization, IGHV1-7 usage and the proportion of ultralong antibodies increased gradually with response, suggesting its importance to immunity against BRD (Safonova et al. 2022).

### IgM and IgG in ultralong CDR H3 cattle antibodies

Ultralong Ig amplicons underwent more isotype switching than canonical Ig amplicons, specifically to the IgG isotype (Fig. 4). While canonical Ig amplicons also exhibited high IgG isotype expression, it was not significantly different from the IgM isotype (Fig. 4). The high counts of IgM may be indicative of cattle B1 cells expressing IgM, compared to those of humans IgG (Stabel et al. 2022). Notably, cattle can make IgM using two different genes, IGHM1 and IGHM2, either separately or in sequence, before class switching (Ma et al. 2016).

Upon stimulation, B cells undergo clonal expansion, and it is more likely for these cells to switch to the IgG isotype. Cattle IgG have three subisotypes, IgG1, IgG2, and IgG3, that are produced by plasma cells in the spleen, lymph nodes, and bone marrow (Symons et al. 1989; Kacskovics and Butler 1996; Rabbani et al. 1997). It would be beneficial to further investigate the proportion of these subisotypes in tissues, as < 10% of bovine IgG molecules have ultralong CDR H3 (Wang et al. 2013; Stanfield et al. 2016). Based on Noble et al. (2023), we could be prominently measuring transcripts of IgG1 and getting limited IgG2 and IgG3 from our immunizations (Noble et al. 2023). This is because IgG2 is an effective opsonin for bovine monocytes to facilitate phagocytosis, while IgG3 is a weak opsonin but is a better fixator of complement (Tizard 2025). The abundance of IgG1 could be possible in cattle for it controls the antibody-dependent cellular cytotoxicity (ADCC) mechanism, which is dependent on this specific subisotype (Noble et al. 2023; Tizard 2025). Further research is necessary to better understand the distribution of IgG subisotype populations with different immunization routes, adjuvants and doses.

### Draining lymph nodes and bone marrow

Specific tissues that expressed the ultralong Igs had significantly higher switched-to-unswitched ratios, including the bone marrow and medial retropharyngeal lymph node (Fig. 5). While the bone marrow had the highest ratio due to a proportionally lower unswitched isotype expression, the medial retropharyngeal lymph node had the highest IgG isotype counts. This tissue was likely a major draining lymph node due to its proximity to the targeted injection sites. IgG amplicon counts in the bone marrow were the next highest, but it wasn’t statistically significant from some other isotype tissue combinations, including IgA in the ileal Peyer’s patch. Observations seen in the bone marrow were interesting because we hypothesized that the IgM isotype would be more prevalent, as the primary site for B cell development. While initial diversification occurs in the bone marrow, we speculate that memory B cells produced by germinal center reactions in the periphery are also returning to this organ.

furtherStatistically significant class-switching was not found in the ileal Peyer’s patch, where SHM can occur before antigen exposure (Liljavirta et al. 2014). In sheep, this is a primary lymphoid organ, where B cells proliferate and the surviving cells are released into circulation (Reynolds 1986; Motyka and Reynolds 1991). The elevated frequency of IgA in the ileal Peyer’s patch could be indicative of B cell proliferation, for positively selected B cells could have circulated and then colonized in other secondary lymphoid tissues. Additionally, it has been suggested that the stimulation from the gut microbiota could promote B cells to diversify (Chen et al. 2020). This supports our findings that counts in the jejunum (IgA), reticulum (IgG), cecum (IgA), spleen (IgG), subiliac lymph node (IgG), mandibular lymph node (IgG), mesenteric lymph node (IgG), medial retropharyngeal lymph node (IgM), and peripheral blood mononuclear cells (IgM/IgG) were statistically equal in the mean counts found in the bone marrow, as shown in Fig. 6b-d. We also observed six tissues that had isotypes dependent on the CDR H3 length, all of which exhibited higher expression of IgM and IgG in canonical Igs (Fig. 6e-k). Interestingly the caudal lobe of the lung and muscle had high expression of these Igs. Among the more dedicated primary and secondary lymphoid tissues, the bone marrow demonstrated similar trends, perhaps indicating that immature B cells and long-lived plasma cells are present. Additionally, the mandibular lymph node, medial retropharyngeal lymph node, and mesenteric lymph node manifested similar patterns, supporting that these tissues may act as a draining lymph node. The higher abundance of canonical CDRH3 is supported by a study by Walther et al. (2013), which showed that IgM and IgG1-3 isotypes have a CDR H3 length of approximately 25.6 and 32.7, respectively, indicating the usage of canonical CDR H3. Regarding the medial retropharyngeal lymph node, while ultralong CDR H3s were present, it can suggest that they are not necessarily a memory focused compared to the canonical CDR H3. As mentioned, this tissue was likely a major draining lymph node, and the hyperimmunization could suggest structural compatibility among the canonical CDR H3. Studies are warranted to investigate differences in cysteine mutations between canonical and ultralong CDR H3 sequences across all tissues to determine variation in knob topology driven by cysteine diversification. Furthermore, analysis identified that CDR H3 length is dependent on tissue, but only within the IgG isotype. The bone marrow showed the highest expression of the IgG isotype, albeit nonsignificant, as counts did not differ significantly from the mandibular lymph node and medial retropharyngeal lymph node. These B cells could differentiate into plasma or memory cells that circulate back to the bone marrow.

### Limitations and future directions

Certain limitations should be considered when interpreting the findings. To continue this study in future directions, SHM analysis could be performed in addition to the inclusion of more cattle of a set age and breed that have undergone routine vaccinations, which would allow all variables to be included in the same model, thus giving a more representative data set. Additional studies could explore changes with age and breed to be most helpful for the producer.

Furthermore, investigating immunization routes would also be necessary to rule out effects on specific draining lymph nodes. To capture the overall immune response, identifying other molecules like cytokines would be of interest. This is because in order for class switching to occur, CD40 on B cells must be signaled in order to bind to CD154 on helper T cells, which activates AID (Durie et al. 1994). Cytokine signaling turns on specific germline promoters, allowing AID to target those switch regions. Namely, IL-4 promoting class switch to IgG1/IgE (Vercelli et al. 1989; Fujieda et al. 1995; Stavnezer 1996; Geha et al. 2003; Cerutti et al. 2005), IFN-γ to IgG2/IgA (Defrance et al. 1992; Stavnezer 1996; Malisan et al. 1996; Lu et al. 1998; Cerutti et al. 2005), and TGF-β to IgA (Defrance et al. 1992; Stavnezer 1996; Cerutti et al. 2005). Innate immune markers would also be interesting for the bovine genome has remarkably large numbers of genes associated with innate immunity (Elsik et al. 2009). Data on PBMCs could be important in creating a standard within vascularized tissues, as this proved to be a major complication in analyzing the results of specific tissues. Probe-based qPCR or digital droplet PCR should also be considered to quantify the gene expression beyond that obtainable through amplicon sequencing of PCR products used in this study, as well as negative selection techniques for cell sorting without manipulation of surface markers. Other techniques, such as laser capture microdissection (LCM) and the expression of Ig by single cells, would enhance our understanding of the bovine immune system. Nevertheless, the results from this study provide a valuable comparison of class-switched ultralong Ig amplicons versus canonical Igs in numerous cattle tissues, namely the BOMA and the draining lymph nodes.

The unique features of cattle ultralong HC provide new avenues of research when considering complex viruses in all species. The development of treatments and preventative measures that effectively mimic the reach (stalk and knob) of the ultralong HC could lead to medical developments in fighting viruses with typically inaccessible or rapidly changing epitopes, such as human immunodeficiency virus (HIV) (Altman, 2024; Sok et al. 2017) and Severe Acute Respiratory Syndrome Coronavirus (SARS-COV-2) (Huang et al. 2023; Tsoleridis et al. 2025). Through experimentation with serum-derived bovine immunoglobulin isolates (SBI), treatment and prevention of childhood malnutrition (Lembcke et al. 1997; Bégin et al. 2008) and gastrointestinal diseases have been observed (Good and Panas 2015; Shafran et al. 2015). SBI is also being used in attempts to treat Irritable Bowel Syndrome (IBS) (Wilson et al. 2013) and HIV-associated enteropathy (Asmuth et al. 2013). Cattle ultralong HC can be used to target challenging epitopes within other species through the creation of monoclonal antibodies. In fact, recent studies have been able to demonstrate how cattle immunized with trimers of antigenic HIV envelope glycoprotein produce an immune response via neutralizing CDR H3 antibodies, clearing the infection (Sok et al. 2017; Huang et al. 2023).

## Conclusion

Little was known about the distribution of ultralong CDR H3s in cattle tissues and our findings demonstrate that ultralong cattle Igs are preferentially switched to the IgG isotype, especially in the medial retropharyngeal lymph node, a draining lymph node. Additionally, B cells producing canonical Igs were found to be more often switched to IgG in the bone marrow. These insights contribute to a better understanding of class switching in both the canonical and ultralong CDR H3, paving the way for future studies aimed at understanding the bovine immune response. Taking these steps will help the community better understand isotype-specific immune responses in cattle tissues, providing a deeper insight into their function within the cattle immune system and aiding in the design of vaccines or treatments for cows and humans.

## Supplementary Information

Below is the link to the electronic supplementary material.

Supplementary Material 1 (DOCX 3.40 MB)

Supplementary Material 2 (XLSX 89.7 MB)

Supplementary Material 3 (XLSX 134 MB)

## Data Availability

The datasets generated and analyzed during the current study are available from the corresponding author on request.

## Abbreviations

ABOM: Abomasum
AID: Activation-induced cytidine deaminase
ADCC: Antibody-dependent cellular cytotoxicity
aa: Amino acids
bp: Base pair
BOMA: Bone marrow
BRAI: Brain
BRD: Bovine respiratory disease
CAUD: Caudal lobe of the lung
CECU: Cecum
COLO: Colon
CDR: Complementarity-determining region
CDR H3: Complementarity-determining region heavy chain 3
CN: Canonical
CRAN: Cranial lobe of the lung
DUOD: Duodenum
ELISA: Enzyme-Linked Immunosorbent Assay
EPLN: Epigastric lymph node
GALL: Gallbladder
HC: Heavy chain
HIV: Human immunodeficiency virus
ILEU: Ileum
ILPP: Ileal Peyer’s patch
IBS: Irritable Bowel Syndrome
JEJU: Jejunum
LC: Light chain
MALN: Mandibular lymph node
MELN: Mesenteric lymph node
MRLN: Medial retropharyngeal lymph node
MUSC: Muscle
OMAS: Omasum
PBMC: Peripheral blood mononuclear cells
RETI: Reticulum
RUME: Rumen
SARS-CoV-2: Severe Acute Respiratory Syndrome Coronavirus
SBI: Serum-derived bovine immunoglobulin isolates
SHM: Somatic hypermutation
SCLN: Superficial cervical lymph node
SPLE: Spleen
SULN: Subiliac lymph node
TdT: Terminal deoxynucleotidyl transferase
TBS: Tris-Buffered Saline
TBST: Tris-Buffered Saline containing 0.1% Tween 20
UL: Ultralong
