# Supplementary material for: Class switched bovine ultralong CDR H3 amplicons versus canonical in immune and non-immune tissues
Source: Immunogenetics. 2026 Feb 23;78(1):3. doi: 10.1007/s00251-026-01395-1 (PMC12926255; doi:10.1007/s00251-026-01395-1)
Supplement: Supplementary file 1 — Supplementary Material 1 (DOCX 3.40 MB) [file 251_2026_1395_MOESM1_ESM.docx]

**Supplemental (In order of discussion)**

**Supplemental Figure 1. IgG titers of both steers exhibited similar patterns following immunization.** ELISA was used to quantify antigen specific IgG responses in PBMCs collected after TIM3 and PD1 immunizations between days 0 through 588 and 565, respectively, for each steer. Values were normalized based on dilution factors for each day indicated in the table for each steer. The corresponding timeline shows when blood was collected (red arrows). Additional arrows represent the immunizations including adjuvants used: RIBI + 500 prime(gray), RIBI boost(blue), Freund’s complete adjuvant boost (FCA; green), and Freund’s incomplete adjuvant boost (IFA: orange). Bar graphs present 450nm absorbance values shown with dilutions corrected to normalize. Dilution factors for each day’s representative absorbance is above the timeline.

**
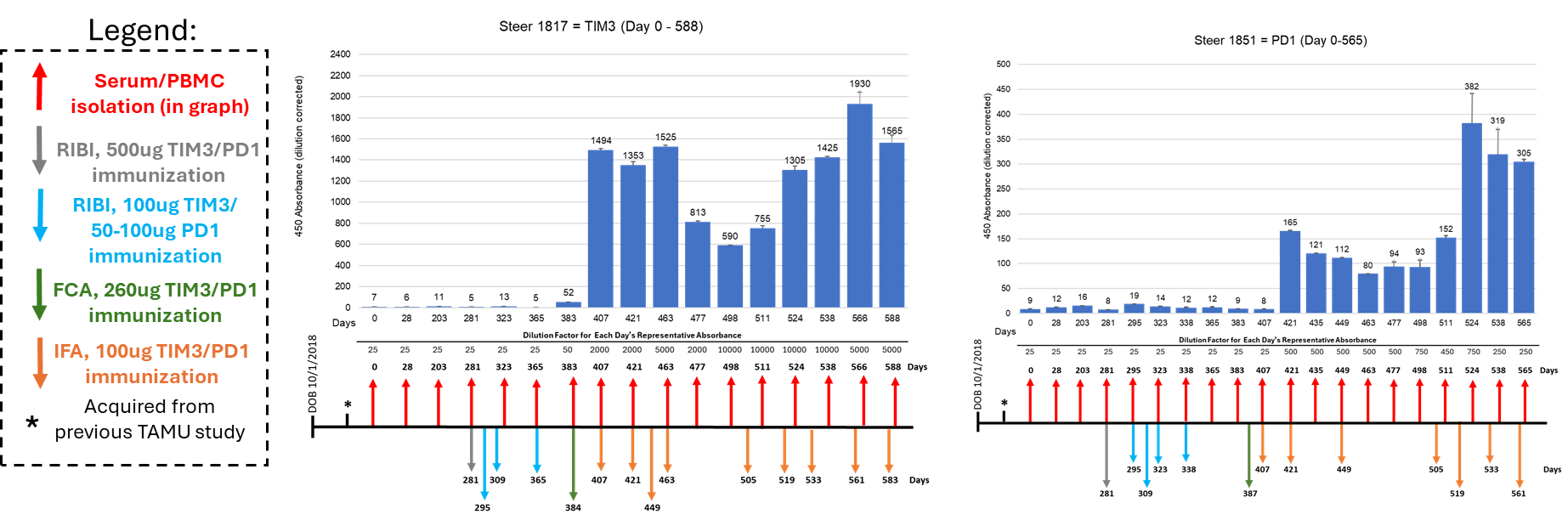
**

**Supplemental Figure 2:** PCR amplification of the PCR product for tissues from steer mix multiplex of testing primers. The PCRs were 10 µl reactions, where 5 µl of that reaction was added to the 1.5% gel well. The electrophoresis run time was 30 seconds on high, then 75 min at 83V to confirm band sizes of IgM/D: 651 bp (common), 513 bp (ultralong), 350 bp (canonical); IgG: 655 bp (common), 517 bp (ultralong), 354bp (canonical); IgA: 655 bp (common), 517 bp (ultralong), 354bp (canonical); IgE: 649 bp (common), 511 bp (ultralong), 348 bp (canonical). Dimers were observed beneath the bands, as expected. Samples were stated left to right:

**
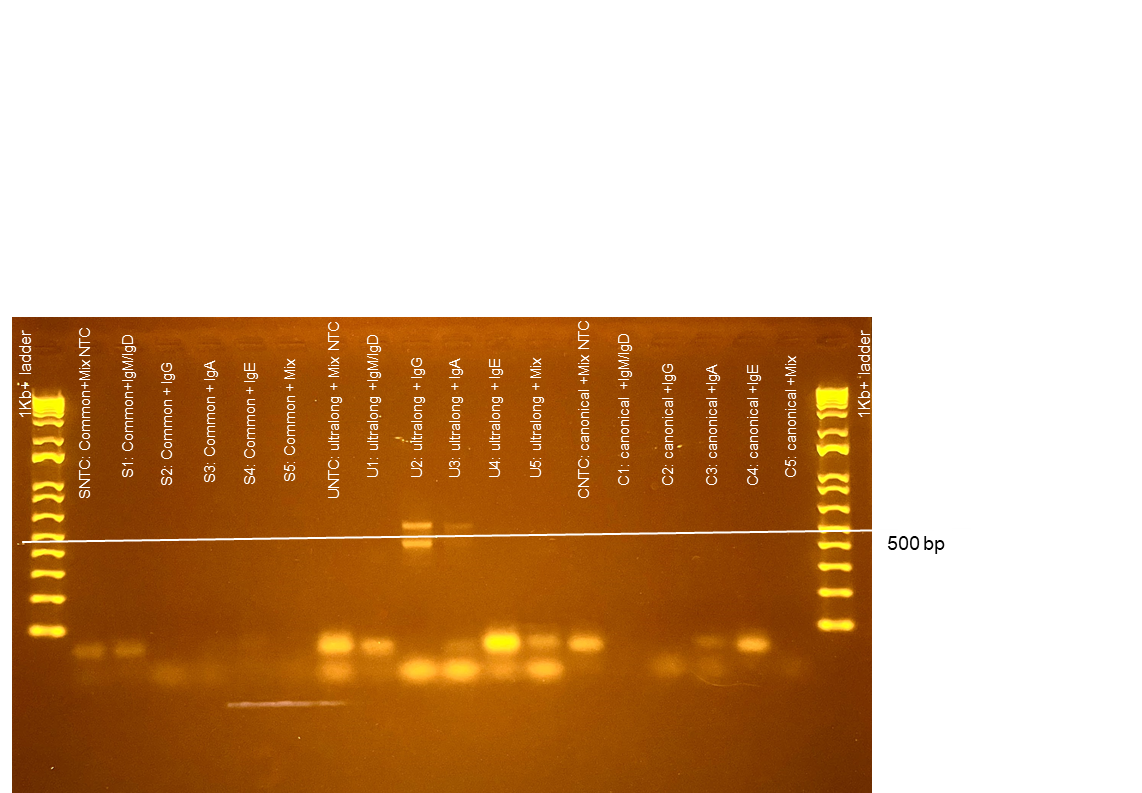
**

**Supplemental Table 1:** **Primers and barcodes for each tissue.** Primers were generated in Geneious Prime 2022.0.1 (https://www.geneious.com) and used throughout the project. The primer name and region are denoted. The direction of the primer is represented by an “F” or an “R” meaning forward and reverse, respectively. The primer sequence consists of an Illumina adapter (red), an eight bp unique barcode that represents each tissue specified in the primer name (bolded), and the primer sequence (black). The Tm is representative of the individual primer melting temperatures in degrees Celsius.

| **Primer** | **F/R** | **Region** | **Sequence** | **Tm** |
| --- | --- | --- | --- | --- |
| GAPDH | F | designed against NM_001034034 | GATGGTGAAGGTCGGAGTGAAC | 60.9 |
| GAPDH | R | designed against NM_001034034 | AGTTGGTGGTGCAGGAGGC | 62.5 |
| VH1-10/VH1-07 common abomasum | F | beginning of FR3 | ACACTCTTTCCCTACACGACGCTCTTCCGATCT**AAGCAAGC**TCCCGGCTCAGCATCACC | 60.5 |
| VH1-10/VH1-07 common bone marrow | F | beginning of FR3 | ACACTCTTTCCCTACACGACGCTCTTCCGATCT**AACCGCTA**TCCCGGCTCAGCATCACC | 60.5 |
| VH1-10/VH1-07 common brain | F | beginning of FR3 | ACACTCTTTCCCTACACGACGCTCTTCCGATCT**AACCATCG**TCCCGGCTCAGCATCACC | 60.5 |
| VH1-10/VH1-07 common cecum | F | beginning of FR3 | ACACTCTTTCCCTACACGACGCTCTTCCGATCT**AATAGCGG**TCCCGGCTCAGCATCACC | 60.5 |
| VH1-10/VH1-07 common colon | F | beginning of FR3 | ACACTCTTTCCCTACACGACGCTCTTCCGATCT**AAGCTTCG**TCCCGGCTCAGCATCACC | 60.5 |
| VH1-10/VH1-07 common duodenum | F | beginning of FR3 | ACACTCTTTCCCTACACGACGCTCTTCCGATCT**AACGTTCG**TCCCGGCTCAGCATCACC | 60.5 |
| VH1-10/VH1-07 common epigastric lymph node | F | beginning of FR3 | ACACTCTTTCCCTACACGACGCTCTTCCGATCT**AACGATGG**TCCCGGCTCAGCATCACC | 60.5 |
| VH1-10/VH1-07 common gallbladder | F | beginning of FR3 | ACACTCTTTCCCTACACGACGCTCTTCCGATCT**AAGGCGTA**TCCCGGCTCAGCATCACC | 60.5 |
| VH1-10/VH1-07 common ileal peyer's patch | F | beginning of FR3 | ACACTCTTTCCCTACACGACGCTCTTCCGATCT**AACCGGAA**TCCCGGCTCAGCATCACC | 60.5 |
| VH1-10/VH1-07 common ileum | F | beginning of FR3 | ACACTCTTTCCCTACACGACGCTCTTCCGATCT**AAGGTACG**TCCCGGCTCAGCATCACC | 60.5 |
| VH1-10/VH1-07 common jejunum | F | beginning of FR3 | ACACTCTTTCCCTACACGACGCTCTTCCGATCT**AATACCGC**TCCCGGCTCAGCATCACC | 60.5 |
| VH1-10/VH1-07 common lung (caudal lobe) | F | beginning of FR3 | ACACTCTTTCCCTACACGACGCTCTTCCGATCT**AACCTAGC**TCCCGGCTCAGCATCACC | 60.5 |
| VH1-10/VH1-07 common lung (cranial lobe) | F | beginning of FR3 | ACACTCTTTCCCTACACGACGCTCTTCCGATCT**AATTCGCG**TCCCGGCTCAGCATCACC | 60.5 |
| VH1-10/VH1-07 common mandibular lymph node | F | beginning of FR3 | ACACTCTTTCCCTACACGACGCTCTTCCGATCT**AACGTAGG**TCCCGGCTCAGCATCACC | 60.5 |
| VH1-10/VH1-07 common medial retropharyngeal lymph node | F | beginning of FR3 | ACACTCTTTCCCTACACGACGCTCTTCCGATCT**AACGAACG**TCCCGGCTCAGCATCACC | 60.5 |
| VH1-10/VH1-07 common mesenteric lymph node | F | beginning of FR3 | ACACTCTTTCCCTACACGACGCTCTTCCGATCT**AACGCCAT**TCCCGGCTCAGCATCACC | 60.5 |
| VH1-10/VH1-07 common muscle | F | beginning of FR3 | ACACTCTTTCCCTACACGACGCTCTTCCGATCT**AACCATGC**TCCCGGCTCAGCATCACC | 60.5 |
| VH1-10/VH1-07 common omasum | F | beginning of FR3 | ACACTCTTTCCCTACACGACGCTCTTCCGATCT**AAGCTACC**TCCCGGCTCAGCATCACC | 60.5 |
| VH1-10/VH1-07 common peripheral blood mononuclear cells | F | beginning of FR3 | ACACTCTTTCCCTACACGACGCTCTTCCGATCT**AACCTTGG**TCCCGGCTCAGCATCACC | 60.5 |
| VH1-10/VH1-07 common reticulum | F | beginning of FR3 | ACACTCTTTCCCTACACGACGCTCTTCCGATCT**AAGGATCG**TCCCGGCTCAGCATCACC | 60.5 |
| VH1-10/VH1-07 common rumen | F | beginning of FR3 | ACACTCTTTCCCTACACGACGCTCTTCCGATCT**AAGCGGTA**TCCCGGCTCAGCATCACC | 60.5 |
| VH1-10/VH1-07 common spleen | F | beginning of FR3 | ACACTCTTTCCCTACACGACGCTCTTCCGATCT**AACCTACG**TCCCGGCTCAGCATCACC | 60.5 |
| VH1-10/VH1-07 common subiliac lymph node | F | beginning of FR3 | ACACTCTTTCCCTACACGACGCTCTTCCGATCT**AATTGCCG**TCCCGGCTCAGCATCACC | 60.5 |
| VH1-10/VH1-07 common superficial cervical lymph node | F | beginning of FR3 | ACACTCTTTCCCTACACGACGCTCTTCCGATCT**AACGCGAA**TCCCGGCTCAGCATCACC | 60.5 |
| IgM/IgD set 1 with P7 adaptor | R | CH1 | GACTGGAGTTCAGACGTGTGCTCTTCCGATCTCCACCGTGCTCTCATCG | 55.0 |
| IgG Tm set 1 with P7 adaptor | R | CH1 | GACTGGAGTTCAGACGTGTGCTCTTCCGATCTCACGGTGGAGCTGGATG | 54.0 |
| IgA Tm set 1 with P7 adaptor | R | CH1 | GACTGGAGTTCAGACGTGTGCTCTTCCGATCTGACCAGGCAGCCGATG | 54.0 |
| Walthers_IgHC-E with P7 adaptor | R | CH1 | GACTGGAGTTCAGACGTGTGCTCTTCCGATCTGCCCAGCCTTACACGGGCTT | 63.0 |

**Supplemental Figure 3:** PCR amplification of the PCR product for tissues from steer 1851 testing barcoded Illumina primers (5µM). The PCRs were 50 µl reactions, where 5 µl of that reaction was added to the 1.0% gel well. The electrophoresis run time was 100 min at 82V to confirm band sizes of 250–350 bp. Dimers were observed beneath the bands, as expected. Samples were stated left to right:

**
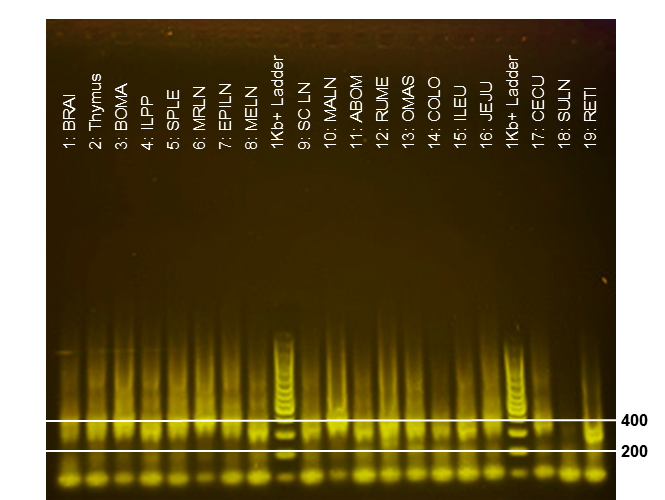
**

**Supplemental Figure 4:** PCR amplification of the PCR product for tissues from each steer for sequencing with barcoded Illumina primers. The PCRs were 50 µl reactions, where 5 µl of that reaction was added to the 1.0% gel well. The electrophoresis run time was 10 seconds on high, followed by 83V on low for 75 minutes to confirm band sizes of 250–330 bp. Dimers were observed beneath the bands, as expected. Samples were stated left to right:

A) Steer 1851 cranial lobe of lung, caudal lobe of lung, subiliac lymph node, gallbladder, muscle; Steer 1817 peripheral blood mononuclear cells; 1851 muscle.

B) Steer 1817 brain, bone marrow, ileal Peyer’s patch, spleen, medial retropharyngeal lymph node; Steer 1851 peripheral blood mononuclear cells; Steer 1817 epigastric lymph node, mesenteric lymph node, superficial cervical lymph node, mandibular lymph node, subiliac lymph node.

C) Steer 1817 abomasum, reticulum, rumen, omasum, colon, gallbladder, jejunum, cecum, duodenum, cranial lobe of lung, caudal lobe of lung.

**
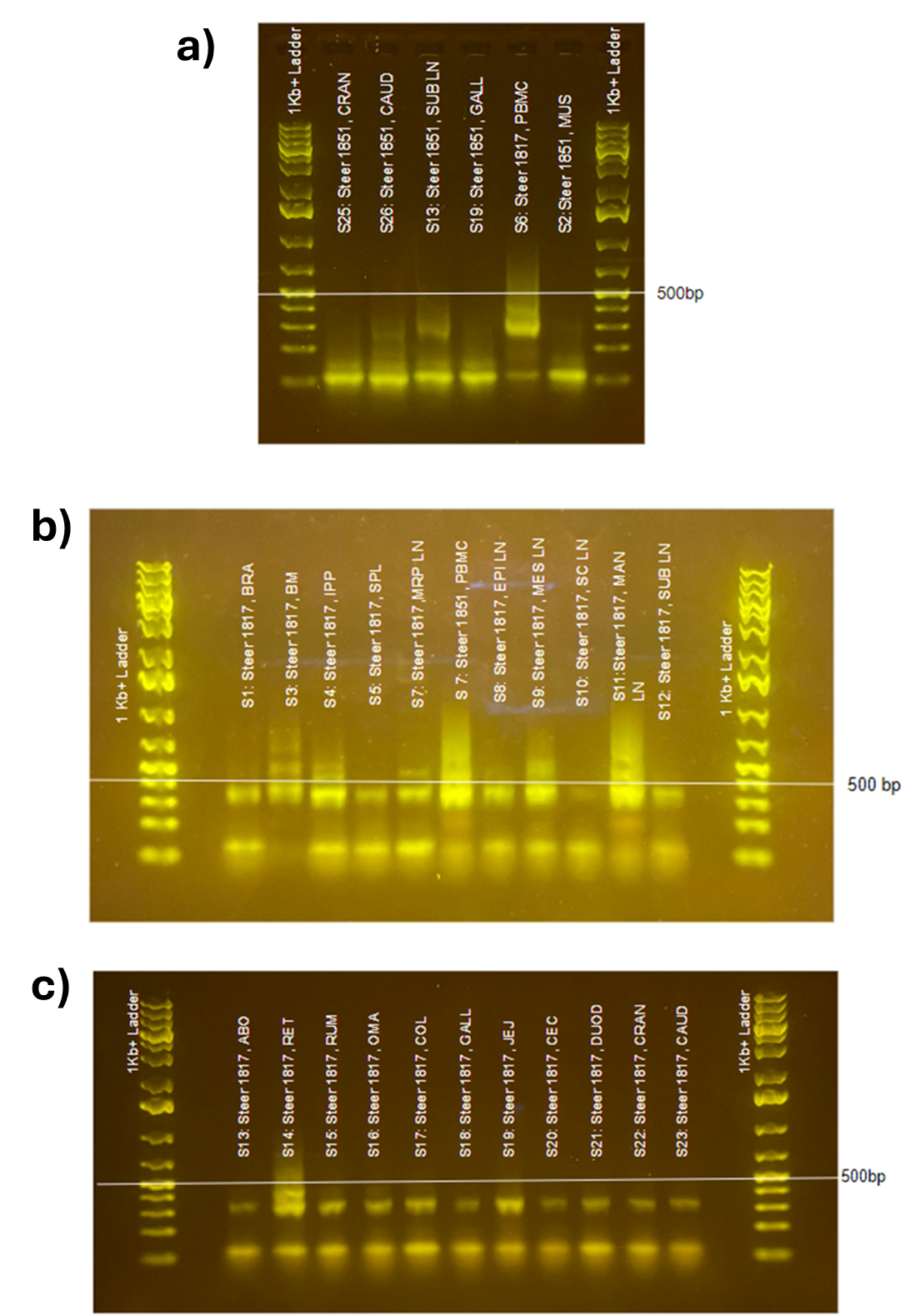
**

**Supplemental Figure 5. Steers share similar patterns of VH, DH, and JH segment usage.** [A] Canonical transcripts incorporated most of the functional VH segments in rearranged sequences. In contrast, nearly all ultralong transcripts utilized VH1-7, the gene segment containing the 8-base pair duplication that encodes the TTVHQ motif (1817; 95.8% and 1851; 94.0%). [B] Canonical and ultralong transcripts integrated most functional DH segments. However, ultralong did utilize more DH8-2 (1817; 37.8% and 1851; 34.1%), which contains a high density of germline encoded cysteines that contribute to the stalk and knob. [C] Canonical and ultralong transcripts utilized the JH2-4 segment, where usage for CN was 97.1% (1817) and 96.9% (1851), while UL was 99.9% (1817) and 100% (1851).

**
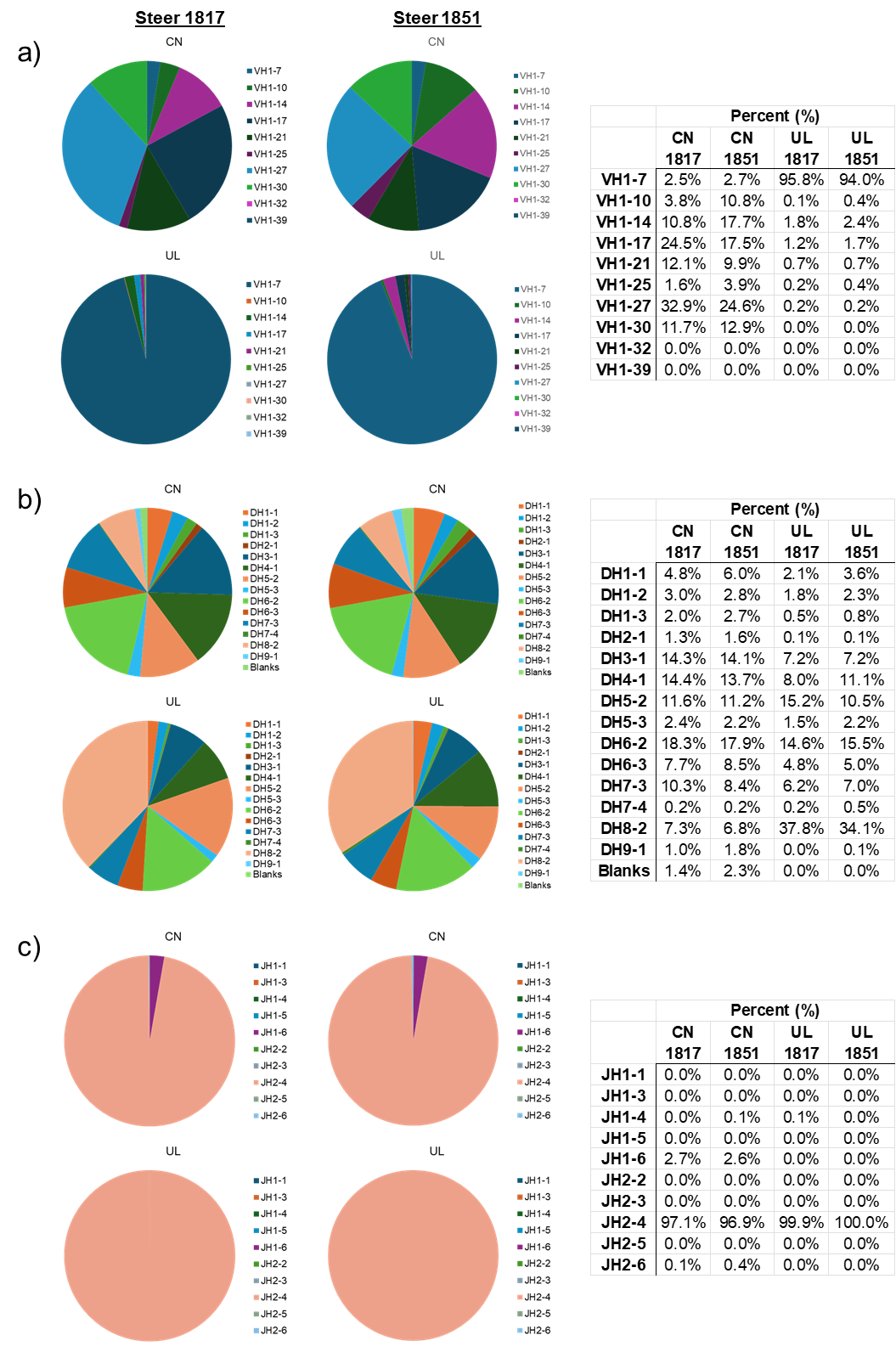
**

**Supplemental Figure 6. Steers share similar isotype proportions.** Among the unswitched isotypes, IgM (red) is proportionally greater than IgD (orange). In the context of the switched isotypes, IgG (green) dominates relatively to IgE (blue) and IgA (red). Overall, IgM and IgG were the overriding isotypes found in canonical transcripts, while most ultralong transcripts were IgG. Canonical class switched isotypes compromised in 60.1% (1817) and 51.1% (1851) of cases, compared to ultralong, which was 76.2% (1817) and 78.1% (1851), respectively.

**
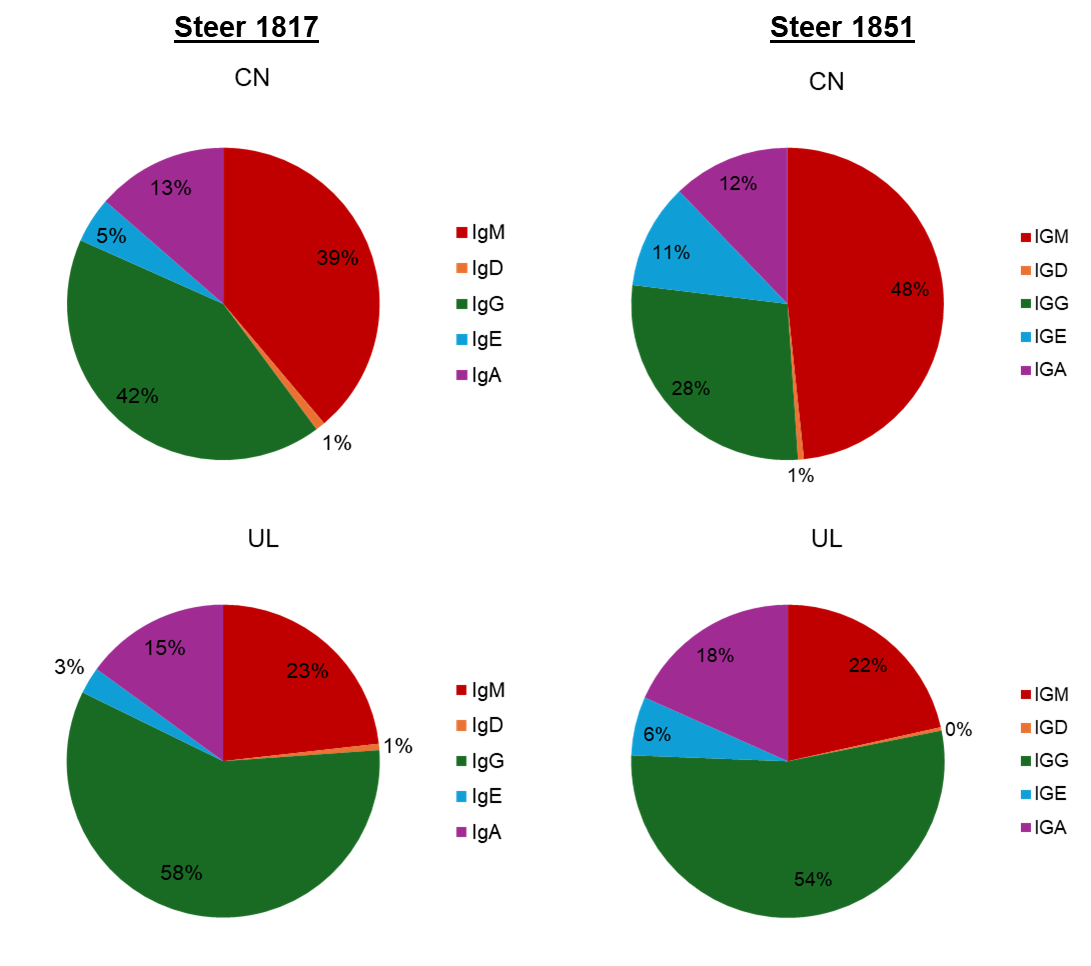
**

**Supplemental Table 2: Unique amplicon counts organized by CDR H3 length, isotype, and tissue from each steer.** The green column contains the total amplicon counts for each CDR H3 length, including all isotypes in each tissue. The last column, in red, contains the total amplicon counts for each tissue, including all isotypes and both CDR H3 lengths. The last row, in purple, contains the total amplicon counts for each isotype, each CDR H3 length, and the grand total for the steer.

| **Steer 1817** | | | | | | | | | | | | | |
| --- | --- | --- | --- | --- | --- | --- | --- | --- | --- | --- | --- | --- | --- |
|  | **CDR H3 Length** | | | | | | | | | | | | |
|  | **CN** | | | | | | **UL** | | | | | |  |
|  | **Isotype** | | | | | | **Isotype** | | | | | |  |
| **Tissue** | **IgM** | **IgD** | **IgG** | **IgE** | **IgA** | **Total CN** | **IgM** | **IgD** | **IgG** | **IgE** | **IgA** | **Total UL** | **TOTAL** |
| ABOM | 185 | 2 | 118 | 12 | 78 | 395 | 2 | 0 | 6 | 4 | 7 | 19 | 414 |
| BOMA | 2713 | 10 | 6142 | 913 | 817 | 10595 | 47 | 0 | 294 | 30 | 14 | 385 | 10980 |
| BRAI | 134 | 5 | 216 | 10 | 46 | 411 | 5 | 1 | 13 | 0 | 3 | 22 | 433 |
| CAUD | 161 | 0 | 88 | 17 | 32 | 298 | 2 | 0 | 4 | 1 | 1 | 8 | 306 |
| CECU | 132 | 2 | 89 | 16 | 32 | 271 | 3 | 0 | 7 | 0 | 1 | 11 | 282 |
| COLO | 367 | 7 | 209 | 31 | 108 | 722 | 6 | 0 | 9 | 2 | 11 | 28 | 750 |
| CRAN | 197 | 3 | 110 | 19 | 28 | 357 | 4 | 0 | 3 | 1 | 0 | 8 | 365 |
| DUOD | 210 | 1 | 135 | 22 | 129 | 497 | 3 | 0 | 8 | 2 | 10 | 23 | 520 |
| EPLN | 533 | 3 | 331 | 56 | 90 | 1013 | 5 | 2 | 19 | 2 | 2 | 30 | 1043 |
| GALL | 152 | 1 | 81 | 17 | 28 | 279 | 2 | 1 | 7 | 0 | 4 | 14 | 293 |
| ILEU | 1 | 1 | 0 | 0 | 0 | 2 | 0 | 0 | 0 | 0 | 0 | 0 | 2 |
| ILPP | 602 | 6 | 406 | 155 | 1809 | 2978 | 30 | 0 | 33 | 3 | 234 | 300 | 3278 |
| JEJU | 627 | 7 | 372 | 68 | 105 | 1179 | 11 | 0 | 20 | 2 | 3 | 36 | 1215 |
| MALN | 4004 | 38 | 4067 | 326 | 748 | 9183 | 76 | 0 | 319 | 10 | 26 | 431 | 9614 |
| MELN | 1429 | 52 | 2247 | 108 | 224 | 4060 | 53 | 2 | 196 | 4 | 20 | 275 | 4335 |
| MRLN | 4388 | 122 | 4514 | 145 | 417 | 9586 | 342 | 8 | 681 | 8 | 24 | 1063 | 10649 |
| MUSC | 53 | 1 | 45 | 4 | 17 | 120 | 1 | 0 | 1 | 1 | 0 | 3 | 123 |
| OMAS | 242 | 0 | 273 | 24 | 52 | 591 | 4 | 0 | 27 | 2 | 7 | 40 | 631 |
| PBMC | 5428 | 317 | 3917 | 698 | 2778 | 13138 | 267 | 9 | 261 | 24 | 139 | 700 | 13838 |
| RETI | 985 | 11 | 934 | 114 | 431 | 2475 | 47 | 3 | 357 | 12 | 76 | 495 | 2970 |
| RUME | 213 | 2 | 289 | 24 | 58 | 586 | 4 | 0 | 14 | 0 | 5 | 23 | 609 |
| SCLN | 46 | 0 | 46 | 5 | 11 | 108 | 0 | 0 | 3 | 0 | 1 | 4 | 112 |
| SPLE | 150 | 0 | 94 | 11 | 22 | 277 | 0 | 0 | 7 | 0 | 1 | 8 | 285 |
| SULN | 238 | 1 | 253 | 34 | 50 | 576 | 0 | 0 | 8 | 1 | 1 | 10 | 586 |
| **TOTAL** | 23190 | 592 | 24976 | 2829 | 8110 | 59697 | 914 | 26 | 2297 | 109 | 590 | 3936 | 63633 |

| **Steer 1851** | | | | | | | | | | | | | | | |
| --- | --- | --- | --- | --- | --- | --- | --- | --- | --- | --- | --- | --- | --- | --- | --- |
| **CDR H3 Length** | | | | | | | | | | | | | | | |
|  | **CN** | | | | | | **UL** | | | | | | |  | |
|  | **Isotype** | | | | | | **Isotype** | | | | | | |  | |
| **Tissue** | **IgM** | **IgD** | **IgG** | **IgE** | **IgA** | **Total CN** | **IgM** | **IgD** | **IgG** | **IgE** | **IgA** | **Total UL** | **TOTAL** | |  |
| ABOM | 1681 | 25 | 534 | 368 | 349 | 2957 | 22 | 3 | 18 | 7 | 11 | 61 | 3018 | |  |
| BOMA | 4020 | 41 | 3733 | 1018 | 951 | 9763 | 57 | 0 | 334 | 39 | 35 | 465 | 10228 | |  |
| BRAI | 2349 | 28 | 707 | 544 | 442 | 4070 | 26 | 0 | 22 | 11 | 16 | 75 | 4145 | |  |
| CAUD | 85 | 0 | 37 | 15 | 12 | 149 | 3 | 0 | 2 | 2 | 0 | 7 | 156 | |  |
| CECU | 2987 | 33 | 1025 | 616 | 1628 | 6289 | 46 | 0 | 61 | 17 | 189 | 313 | 6602 | |  |
| COLO | 1397 | 11 | 256 | 336 | 305 | 2305 | 12 | 0 | 16 | 7 | 7 | 42 | 2347 | |  |
| CRAN | 43 | 0 | 23 | 7 | 6 | 79 | 0 | 0 | 0 | 0 | 0 | 0 | 79 | |  |
| DUOD | 62 | 1 | 27 | 5 | 33 | 128 | 1 | 0 | 2 | 1 | 1 | 5 | 133 | |  |
| EPLN | 1451 | 10 | 1479 | 245 | 276 | 3461 | 32 | 0 | 151 | 5 | 14 | 202 | 3663 | |  |
| GALL | 63 | 0 | 27 | 9 | 3 | 102 | 1 | 0 | 4 | 0 | 0 | 5 | 107 | |  |
| ILEU | 2232 | 25 | 597 | 580 | 623 | 4057 | 17 | 2 | 27 | 6 | 29 | 81 | 4138 | |  |
| ILPP | 3168 | 33 | 1476 | 838 | 727 | 6242 | 31 | 0 | 65 | 16 | 30 | 142 | 6384 | |  |
| JEJU | 1373 | 14 | 614 | 556 | 1329 | 3886 | 27 | 1 | 58 | 24 | 205 | 315 | 4201 | |  |
| MALN | 2060 | 11 | 2380 | 358 | 404 | 5213 | 74 | 0 | 263 | 10 | 14 | 361 | 5574 | |  |
| MELN | 3031 | 37 | 1165 | 659 | 605 | 5497 | 20 | 1 | 42 | 14 | 14 | 91 | 5588 | |  |
| MRLN | 3786 | 97 | 4193 | 371 | 660 | 9107 | 237 | 0 | 445 | 7 | 33 | 722 | 9829 | |  |
| MUSC | 67 | 1 | 49 | 9 | 6 | 132 | 0 | 0 | 1 | 0 | 0 | 1 | 133 | |  |
| OMAS | 2224 | 26 | 729 | 547 | 498 | 4024 | 18 | 1 | 27 | 11 | 12 | 69 | 4093 | |  |
| PBMC | 552 | 33 | 140 | 35 | 77 | 837 | 47 | 0 | 33 | 4 | 31 | 115 | 952 | |  |
| RETI | 4109 | 39 | 1196 | 1015 | 872 | 7231 | 44 | 3 | 47 | 13 | 36 | 143 | 7374 | |  |
| RUME | 3177 | 37 | 1272 | 838 | 653 | 5977 | 28 | 1 | 48 | 17 | 27 | 121 | 6098 | |  |
| SCLN | 1415 | 19 | 538 | 347 | 288 | 2607 | 21 | 1 | 29 | 7 | 13 | 71 | 2678 | |  |
| SPLE | 2718 | 26 | 2418 | 533 | 474 | 6169 | 77 | 1 | 282 | 12 | 17 | 389 | 6558 | |  |
| SULN | 839 | 12 | 1384 | 267 | 88 | 2590 | 23 | 0 | 182 | 14 | 2 | 221 | 2811 | |  |
| **TOTAL** | 44889 | 559 | 25999 | 10116 | 11309 | 92872 | 864 | 14 | 2159 | 244 | 736 | 4017 | 96889 | |  |

**Supplemental Table 2. Steers show similar patterns of amplicon proportions across CDR H3 lengths.** Proportions were calculated for each tissue by taking amplicon counts for each isotype within the CDR H3 length, then dividing by the total amplicon counts for that tissue.

| **Steer 1817** | | | | | | | | | | |
| --- | --- | --- | --- | --- | --- | --- | --- | --- | --- | --- |
|  | **Canonical CDR H3** | | | | | **Ultralong CDR H3** | | | | |
| **Tissue** | **IgM** | **IgD** | **IgG** | **IgE** | **IgA** | **IgM** | **IgD** | **IgG** | **IgE** | **IgA** |
| ABOM | 44.69 | 0.48 | 28.50 | 2.90 | 18.84 | 0.48 | 0.00 | 1.45 | 0.97 | 1.69 |
| BOMA | 24.71 | 0.09 | 55.94 | 8.32 | 7.44 | 0.43 | 0.00 | 2.68 | 0.27 | 0.13 |
| BRAI | 30.95 | 1.15 | 49.88 | 2.31 | 10.62 | 1.15 | 0.23 | 3.00 | 0.00 | 0.69 |
| CAUD | 52.61 | 0.00 | 28.76 | 5.56 | 10.46 | 0.65 | 0.00 | 1.31 | 0.33 | 0.33 |
| CECU | 46.81 | 0.71 | 31.56 | 5.67 | 11.35 | 1.06 | 0.00 | 2.48 | 0.00 | 0.35 |
| COLO | 48.93 | 0.93 | 27.87 | 4.13 | 14.40 | 0.80 | 0.00 | 1.20 | 0.27 | 1.47 |
| CRAN | 53.97 | 0.82 | 30.14 | 5.21 | 7.67 | 1.10 | 0.00 | 0.82 | 0.27 | 0.00 |
| DUOD | 40.38 | 0.19 | 25.96 | 4.23 | 24.81 | 0.58 | 0.00 | 1.54 | 0.38 | 1.92 |
| EPLN | 51.10 | 0.29 | 31.74 | 5.37 | 8.63 | 0.48 | 0.19 | 1.82 | 0.19 | 0.19 |
| GALL | 51.88 | 0.34 | 27.65 | 5.80 | 9.56 | 0.68 | 0.34 | 2.39 | 0.00 | 1.37 |
| ILEU | 50.00 | 50.00 | 0.00 | 0.00 | 0.00 | 0.00 | 0.00 | 0.00 | 0.00 | 0.00 |
| ILPP | 18.36 | 0.18 | 12.39 | 4.73 | 55.19 | 0.92 | 0.00 | 1.01 | 0.09 | 7.14 |
| JEJU | 51.60 | 0.58 | 30.62 | 5.60 | 8.64 | 0.91 | 0.00 | 1.65 | 0.16 | 0.25 |
| MALN | 41.65 | 0.40 | 42.30 | 3.39 | 7.78 | 0.79 | 0.00 | 3.32 | 0.10 | 0.27 |
| MELN | 32.96 | 1.20 | 51.83 | 2.49 | 5.17 | 1.22 | 0.05 | 4.52 | 0.09 | 0.46 |
| MRLN | 41.21 | 1.15 | 42.39 | 1.36 | 3.92 | 3.21 | 0.08 | 6.39 | 0.08 | 0.23 |
| MUSC | 43.09 | 0.81 | 36.59 | 3.25 | 13.82 | 0.81 | 0.00 | 0.81 | 0.81 | 0.00 |
| OMAS | 38.35 | 0.00 | 43.26 | 3.80 | 8.24 | 0.63 | 0.00 | 4.28 | 0.32 | 1.11 |
| PBMC | 39.23 | 2.29 | 28.31 | 5.04 | 20.08 | 1.93 | 0.07 | 1.89 | 0.17 | 1.00 |
| RETI | 33.16 | 0.37 | 31.45 | 3.84 | 14.51 | 1.58 | 0.10 | 12.02 | 0.40 | 2.56 |
| RUME | 34.98 | 0.33 | 47.45 | 3.94 | 9.52 | 0.66 | 0.00 | 2.30 | 0.00 | 0.82 |
| SCLN | 41.07 | 0.00 | 41.07 | 4.46 | 9.82 | 0.00 | 0.00 | 2.68 | 0.00 | 0.89 |
| SPLE | 52.63 | 0.00 | 32.98 | 3.86 | 7.72 | 0.00 | 0.00 | 2.46 | 0.00 | 0.35 |
| SULN | 40.61 | 0.17 | 43.17 | 5.80 | 8.53 | 0.00 | 0.00 | 1.37 | 0.17 | 0.17 |
|  |  |  |  |  |  |  |  |  |  |  |
| **Steer 1851** | | | | | | | | | | |
|  | **Canonical CDR H3** | | | | | **Ultralong CDR H3** | | | | |
| **Tissue** | **IgM** | **IgD** | **IgG** | **IgE** | **IgA** | **IgM** | **IgD** | **IgG** | **IgE** | **IgA** |
| ABOM | 55.70 | 0.83 | 17.69 | 12.19 | 11.56 | 0.73 | 0.10 | 0.60 | 0.23 | 0.36 |
| BOMA | 39.30 | 0.40 | 36.50 | 9.95 | 9.30 | 0.56 | 0.00 | 3.27 | 0.38 | 0.34 |
| BRAI | 56.67 | 0.68 | 17.06 | 13.12 | 10.66 | 0.63 | 0.00 | 0.53 | 0.27 | 0.39 |
| CAUD | 54.49 | 0.00 | 23.72 | 9.62 | 7.69 | 1.92 | 0.00 | 1.28 | 1.28 | 0.00 |
| CECU | 45.24 | 0.50 | 15.53 | 9.33 | 24.66 | 0.70 | 0.00 | 0.92 | 0.26 | 2.86 |
| COLO | 59.52 | 0.47 | 10.91 | 14.32 | 13.00 | 0.51 | 0.00 | 0.68 | 0.30 | 0.30 |
| CRAN | 54.43 | 0.00 | 29.11 | 8.86 | 7.59 | 0.00 | 0.00 | 0.00 | 0.00 | 0.00 |
| DUOD | 46.62 | 0.75 | 20.30 | 3.76 | 24.81 | 0.75 | 0.00 | 1.50 | 0.75 | 0.75 |
| EPLN | 39.61 | 0.27 | 40.38 | 6.69 | 7.53 | 0.87 | 0.00 | 4.12 | 0.14 | 0.38 |
| GALL | 58.88 | 0.00 | 25.23 | 8.41 | 2.80 | 0.93 | 0.00 | 3.74 | 0.00 | 0.00 |
| ILEU | 53.94 | 0.60 | 14.43 | 14.02 | 15.06 | 0.41 | 0.05 | 0.65 | 0.14 | 0.70 |
| ILPP | 49.62 | 0.52 | 23.12 | 13.13 | 11.39 | 0.49 | 0.00 | 1.02 | 0.25 | 0.47 |
| JEJU | 32.68 | 0.33 | 14.62 | 13.23 | 31.64 | 0.64 | 0.02 | 1.38 | 0.57 | 4.88 |
| MALN | 36.96 | 0.20 | 42.70 | 6.42 | 7.25 | 1.33 | 0.00 | 4.72 | 0.18 | 0.25 |
| MELN | 54.24 | 0.66 | 20.85 | 11.79 | 10.83 | 0.36 | 0.02 | 0.75 | 0.25 | 0.25 |
| MRLN | 38.52 | 0.99 | 42.66 | 3.77 | 6.71 | 2.41 | 0.00 | 4.53 | 0.07 | 0.34 |
| MUSC | 50.38 | 0.75 | 36.84 | 6.77 | 4.51 | 0.00 | 0.00 | 0.75 | 0.00 | 0.00 |
| OMAS | 54.34 | 0.64 | 17.81 | 13.36 | 12.17 | 0.44 | 0.02 | 0.66 | 0.27 | 0.29 |
| PBMC | 57.98 | 3.47 | 14.71 | 3.68 | 8.09 | 4.94 | 0.00 | 3.47 | 0.42 | 3.26 |
| RETI | 55.72 | 0.53 | 16.22 | 13.76 | 11.83 | 0.60 | 0.04 | 0.64 | 0.18 | 0.49 |
| RUME | 52.10 | 0.61 | 20.86 | 13.74 | 10.71 | 0.46 | 0.02 | 0.79 | 0.28 | 0.44 |
| SCLN | 52.84 | 0.71 | 20.09 | 12.96 | 10.75 | 0.78 | 0.04 | 1.08 | 0.26 | 0.49 |
| SPLE | 41.45 | 0.40 | 36.87 | 8.13 | 7.23 | 1.17 | 0.02 | 4.30 | 0.18 | 0.26 |
| SULN | 29.85 | 0.43 | 49.24 | 9.50 | 3.13 | 0.82 | 0.00 | 6.47 | 0.50 | 0.07 |
